# Metabolic changes of Interleukin-12/15/18-stimulated human NK cells

**DOI:** 10.1038/s41598-021-85960-6

**Published:** 2021-03-19

**Authors:** Iñigo Terrén, Ane Orrantia, Alba Mosteiro, Joana Vitallé, Olatz Zenarruzabeitia, Francisco Borrego

**Affiliations:** 1 Immunopathology Group, Biocruces Bizkaia Health Research Institute, Barakaldo, Spain; 2 Basque Foundation for Science, Bilbao, Spain

**Keywords:** NK cells, Interleukins, Innate lymphoid cells

## Abstract

Natural Killer (NK) cells acquire memory-like properties following a brief stimulation with IL-12, IL-15 and IL-18. These IL-12/15/18-preactivated NK cells, also known as cytokine-induced memory-like (CIML) NK cells, have been revealed as a powerful tool in cancer immunotherapy due to their persistence in the host and their increased effector functions. Several studies have shown that NK cells modulate their metabolism in response to cytokine-stimulation and other stimuli, suggesting that there is a link between metabolism and cellular functions. In this paper, we have analyzed metabolic changes associated to IL-12/15/18-stimulation and the relevance of glycolytic pathway for NK cell effector functions. We have found CIML NK cells are able to retain a metabolic profile shifted towards glycolysis seven days after cytokine withdrawal. Furthermore, we found that treatment with 2-DG differently affects distinct NK cell effector functions and is stimuli-dependent. These findings may have implications in the design of NK cell-based cancer immunotherapies.

## Introduction

Natural Killer (NK) cells have the ability to directly kill tumor and virus-infected cells without prior sensitization. They can also release a wide variety of cytokines and chemokines that promote an adaptive immune response against target cells. Thus, NK cells represent a valuable tool in cancer immunotherapy, and several strategies have been proposed to exploit and improve their anti-tumor mechanisms in different cancers^1–5^. Among them, a promising therapeutic approach is to stimulate NK cells with interleukins (ILs). For instance, IL-15 and superagonists, such as ALT-803, administration has been proven to be relatively safe and to effectively promote NK cell expansion in cancer patients^6–9^. Alternatively, cells could be activated ex vivo prior to infusion. For example, IL-15-stimulated NK cells have been used in adoptive cell therapy protocols to treat different malignancies^10, 11^. Moreover, NK cells could be stimulated with combinations of ILs to enhance multiple effector functions. An example of a successful therapy following this strategy is the administration of IL-12/15/18-preactivated NK cells, also known as cytokine-induced memory-like (CIML) NK cells. These cells have been proven to be useful in mouse and rat models of several malignancies, including acute myeloid leukemia, T-cell acute lymphoblastic leukemia, multiple myeloma, lymphoma, melanoma, ovarian cancer and hepatocellular carcinoma^12–18^. Regarding to humans, CIML NK cells have shown their safety and efficacy in the treatment of acute myeloid leukemia patients^12, 19^, and are currently being tested in several clinical trials (NCT01898793, NCT02782546, NCT03068819, NCT04024761, NCT04290546, NCT04354025 and NCT04634435, from clinicaltrials.gov).

NK cells have been traditionally defined as innate lymphocytes, although this classification has become more challenging since adaptive NK cells were described in mice, non-human primates and humans^20^. Unlike adaptive NK cells, CIML NK cells have not shown responses to specific antigens, although they share some features of immunological memory. These IL-12/15/18-preactivated NK cells were initially described in mice and were defined by their increased interferon gamma (IFNγ) production in response to a restimulation after a resting period^21^. Moreover, these cells exhibited enhanced persistence in vivo and could be detected up to three months after adoptive transfer^13, 16^. Further studies revealed similar behavior of human IL-12/15/18-stimulated NK cells, showing an enhanced cytokine production following restimulation^12, 14, 22–26^, and increased proliferation^12, 14, 22^. After IL-12/15/18-stimulation, the phenotype of NK cells includes changes in the expression of activating and inhibitory receptors, and chemokine and cytokine receptors^12, 14, 22, 24, 27–29^. Interestingly, it has been also demonstrated that cytokine-stimulation modifies the metabolic activity of NK cells^30, 31^, although this aspect has been poorly explored in human IL-12/15/18-stimulated NK cells.

Immune responses often involve changes in cellular metabolism, which is necessary to fulfill the different energetic demands of each cell function. Specifically related to NK cells, there is mounting evidence that the metabolic profile is modified during cell development, viral infection, and activation. This metabolic reprogramming is necessary to support processes such as IFNγ production^32^, and includes changes in the activity of metabolic regulators, expression of nutrient transporters, and reconfiguration of the metabolic pathways^33^. In this sense, it has been demonstrated that glucose metabolism is crucial for NK cell-mediated control of mouse cytomegalovirus infection^34^. Glycolytic pathway is also essential in mouse NK cells for IFNγ and granzyme B production, although the mechanisms that regulate each function could be different^35^. Certain pathologies such as obesity and cancer lead to altered metabolic activity, which has been linked to dysfunctional NK cells^36–38^. Therefore, current knowledge suggests that there is a close relationship between NK cell metabolism and their effector functions.

Here, we have studied how human NK cell metabolism is modulated following IL-12/15/18-stimulation. Furthermore, considering that these preactivated NK cells have memory-like properties, and that they are being successfully used in clinical trials, we asked if the metabolic changes found immediately after the stimulation persisted for a long time. Since IL-15 and IL-2 have been previously used to promote NK cell survival and expansion, we tested the effect of both ILs after the preactivation with IL-12/15/18. Moreover, we have explored the different glycolytic requirements of several effector functions of IL-12/15/18-preactivated NK cells in response to different stimuli.

## Results

### IL-12/15/18-stimulated and CIML NK cells exhibit increased expression of nutrient transporters

**Figure 1:**
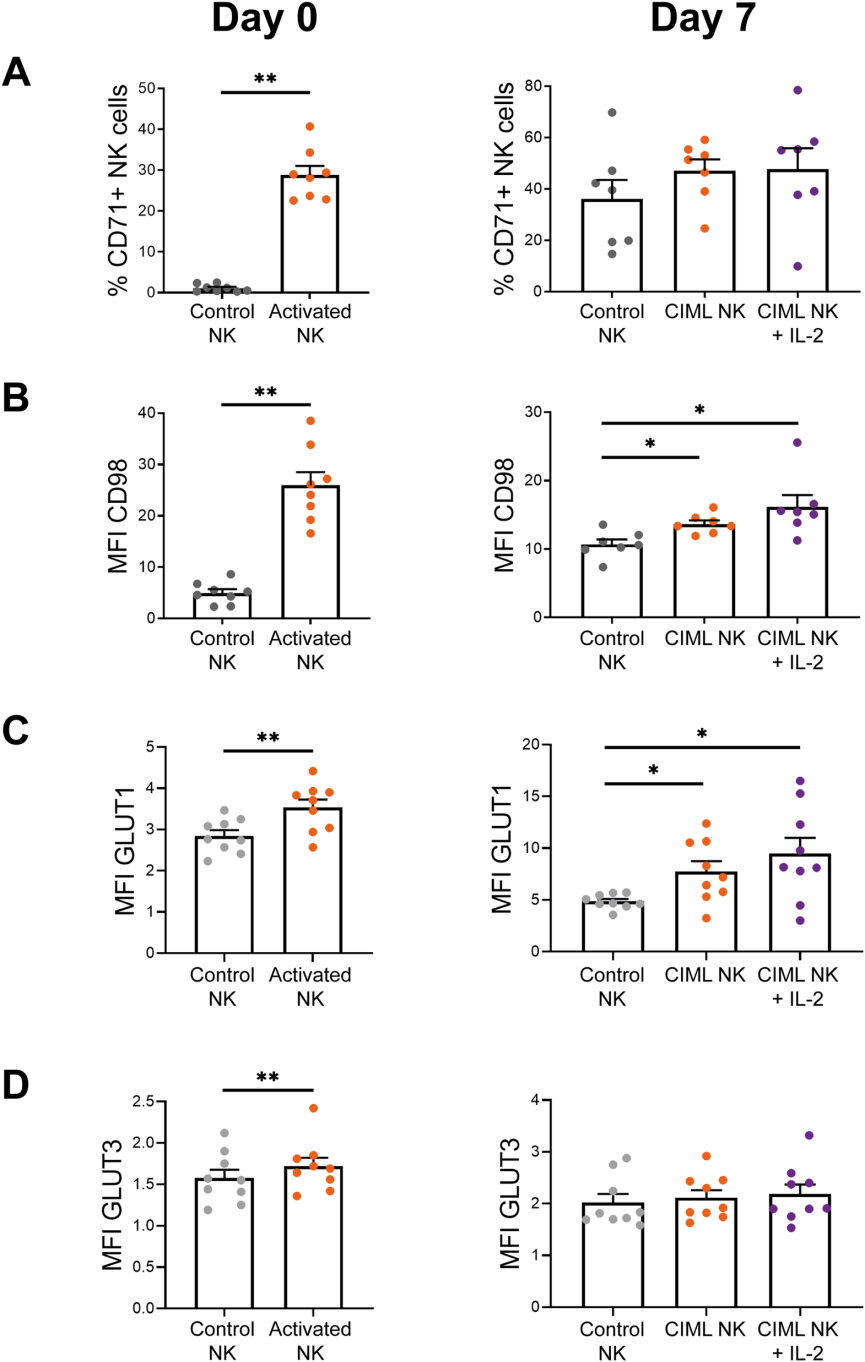
Expression of nutrient transporters in human IL-12/15/18-stimulated NK cells. Bar charts representing (A) the percentage of control NK and IL-12/15/18-stimulated (activated NK or CIML NK at day 0 or 7, respectively) NK cells expressing the transferrin receptor CD71, and expression levels of (B) the heavy chain of multiple heterodimeric amino acid transporters CD98, and glucose transporters (C) GLUT1 and (D) GLUT3, measured as median fluorescence intensity (MFI). Graphs show data of cells after 16–18 h of IL-12/15/18-stimulation (Day 0, left column) and after 7 days of culture with IL-15 (control NK or CIML NK) or IL-2 (CIML NK + IL-2) (Day 7, right column). Means ± SEM are depicted. Statistical analyses were performed using Wilcoxon matched-pairs signed rank test. Each dot represents an independent experiment from a different donor (n = 6–9). *P < 0.05, **P < 0.01.

Previous studies have shown that NK cells can modify the expression of nutrient transporters following cytokine-stimulation^32, 39–46^. In accordance, we have found that NK cells increase the expression of the transferrin receptor CD71, the heavy subunit of multiple heterodimeric amino acid transporters CD98, and the glucose transporters GLUT1 and GLUT3, following stimulation with IL-12, IL-15 and IL-18 (hereinafter referred to as activated NK cells) for 16–18 h, i.e. at day 0 (Fig. 1, left column). In accordance with other reports^32, 46, 47^, we found that CD56^bright^ and CD56^dim^ NK cell subsets exhibit different expression of nutrient transporters. Interestingly, we found that among activated NK cells, CD56^bright^ subset express higher levels of CD71, CD98 and GLUT1 (Supplementary Fig. 1). Of note, GLUT3 is expressed at very low levels and, although statistically significant, very modest increment was detected and may probably not be biologically relevant.

We next asked if the increased expression of nutrient transporters returned to resting levels after cytokine withdrawal, or if it was maintained when cells returned to a less activated state. We cultured control and activated NK cells with low doses of IL-15 (1 ng/mL) for seven days, as others have previously done^12, 26^, to support their survival without inducing a strong activation. By doing so, we expected that cells could return to a resting situation. Furthermore, IL-12/15/18-preactivated NK cells have an increased expression of alpha chain of the IL-2 receptor (IL-2Rα, also known as CD25), which conforms the high-affinity IL-2 receptor^12–14, 22, 24, 27, 28^. This feature allows IL-12/15/18-preactivated NK cells to proliferate in response to low doses of IL-2, as we and others have previously described^13, 22, 24^. Therefore, we washed control and activated NK cells, cultured them for a period of seven days with low concentrations of IL-15 or IL-2 (Supplementary Fig. 2), and then analyzed again the expression of nutrient transporters. Hereinafter, activated NK cells that have been cultured for 7 days will be referred to as CIML NK cells. Our results showed that CIML NK cells express higher levels of CD98 and GLUT1 than control NK cells at day 7 (Fig. 1, right column). On the other hand, no significant differences were found in the expression of CD71 and GLUT3 between control and CIML NK cells (Fig. 1, right column). Of note, CD71 is not (or very low) expressed in resting NK cells at day 0, but culturing them for seven days with low doses of IL-15 was enough to induce CD71 expression (Fig. 1 and Supplementary Fig. 3). Altogether, these results show that NK cells increase the expression of nutrient transporters following stimulation with IL-12, IL-15 and IL-18, and that CIML NK cells preserve an elevated expression of amino acid and glucose transporters when they are maintained in culture with very low concentrations of IL-2 or IL-15.

**Figure 2:**
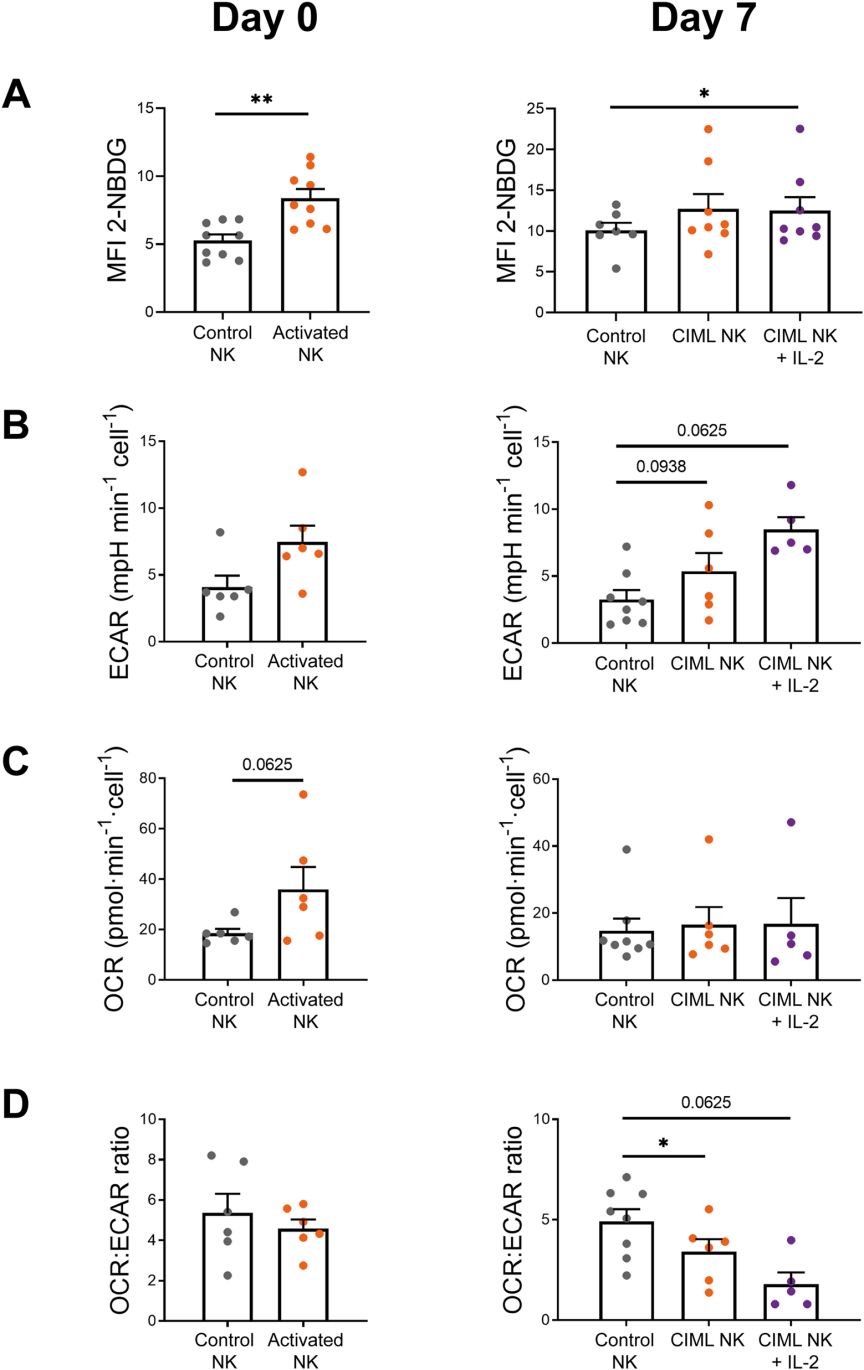
Metabolic switch towards glycolysis of human IL-12/15/18-stimulated NK cells. Bar charts representing (A) glucose analogue 2-NBDG uptake, measured as median fluorescence intensity (MFI). Bar charts representing (B) glycolytic rate, measured as extracellular acidification rate (ECAR); (C) OXPHOS rate, measured as oxygen consumption rate (OCR); and (D) OCR:ECAR ratio. Graphs show data of control NK cells and IL-12/15/18-stimulated NK cells (activated NK) (Day 0, left column), and after 7 days of culture with IL-15 (control NK or CIML NK) or IL-2 (CIML NK + IL-2) (Day 7, right column). Means ± SEM are depicted. Statistical analyses were performed using Wilcoxon matched-pairs signed rank test. Each dot represents an independent experiment from a different donor (n = 5–9). *P < 0.05, **P < 0.01.

**Figure 3:**
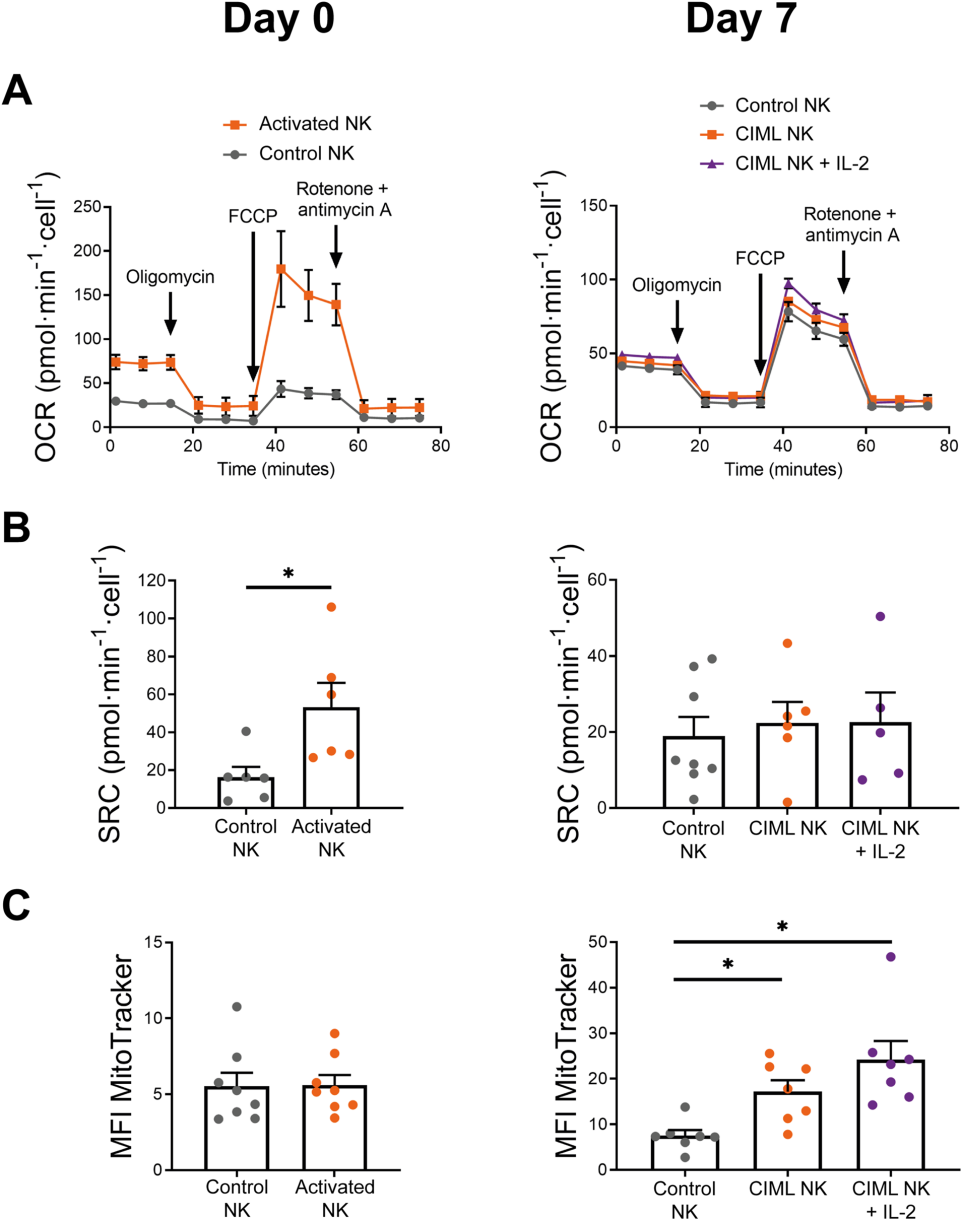
Mitochondrial activity and mass of human IL-12/15/18-stimulated NK cells. (A) Representative experiment of Seahorse XF analyzer with the Mito Stress Test kit. Means ± standard deviation are depicted. (B) Bar charts representing spare respiratory capacity (SRC), measured as the difference between maximal and basal mitochondrial respirations. (C) Bar charts representing mitochondrial mass, measured as the median fluorescence intensity (MFI) of MitoTracker Green. Graphs show data of control NK cells and IL-12/15/18-stimulated NK cells (activated NK) (Day 0, left column), and after 7 days of culture with IL-15 (control NK or CIML NK) or IL-2 (CIML NK + IL-2) (Day 7, right column). Means ± SEM are depicted in bar graphs. Statistical analyses were performed using Wilcoxon matched-pairs signed rank test. Each dot represents an independent experiment from a different donor (n = 5–8). *P < 0.05.

### CIML NK cells retain a metabolic profile shifted towards glycolysis

Considering that CIML NK cells have an increased GLUT1 expression, and that glycolysis supports multiple NK cell functions^33, 38^, we next analyzed changes in this metabolic pathway. Experiments with the glucose analogue 2-NBDG revealed that 2-NBDG uptake is higher in activated and CIML NK cells at days 0 and 7, respectively (Fig. 2A). We further studied glycolytic activity of NK cells with the extracellular flux analyzer Seahorse XF. Our results showed that activated NK cells tended to increase the glycolytic rate, measured as extracellular acidification rate (ECAR) (Fig. 2B, left panel, and Supplementary Fig. 4). These results support the hypothesis that NK cells increase glycolytic activity following stimulation^33^. Interestingly, CIML NK cells tend to show a higher glycolytic rate than control NK cells at day 7, especially when cultured with low doses of IL-2 (Fig. 2B, right panel, and Supplementary Fig. 4). Oxidative phosphorylation (OXPHOS) levels, measured as oxygen consumption rate (OCR), also tended to increase in activated NK cells. However, the differences on OXPHOS levels between control and CIML NK cells were negligible at day 7 (Fig. 2C). Then, we calculated the ratio between OXPHOS and glycolytic rates, which has been previously used to detect shifts in fuel utilization^48^. Our results showed that activated NK cells tended to have a lower OCR:ECAR ratio, although the difference is better observed after seven days, indicating that CIML NK cells undergo a metabolic switch towards glycolysis that is not stopped following cytokine withdrawal (Fig. 2D). Altogether, these results suggest that IL-12/15/18-stimulated NK cells increase their glycolytic machinery, and that they can retain somehow the metabolic profile towards glycolysis for at least seven days when cultured with low concentrations of IL-15 or IL-2.

**Figure 4:**
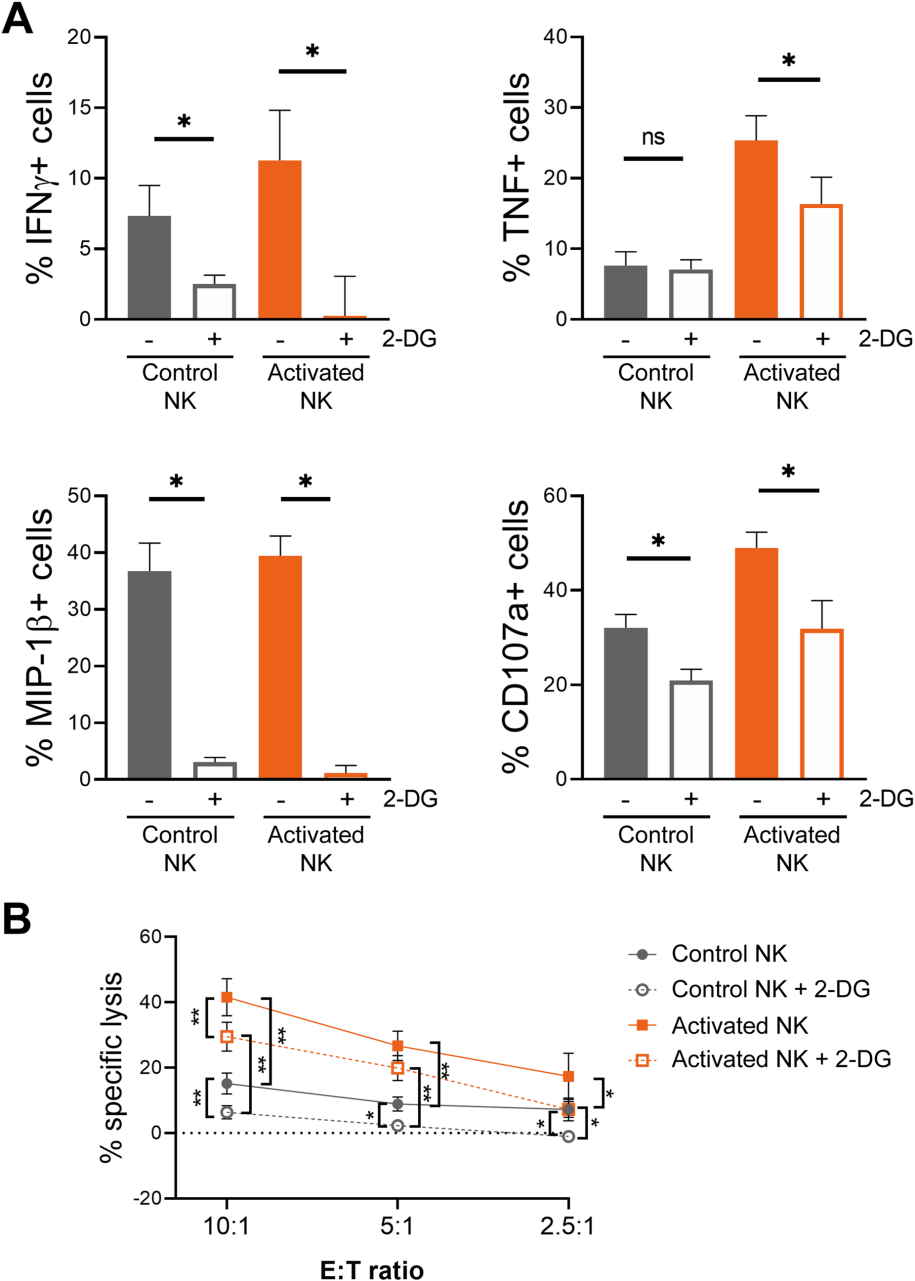
2-DG-induced inhibition of specific cytokine/chemokine production, degranulation and cytotoxic activity of human IL-12/15/18-stimulated NK cells at day 0. (A) Control NK cells and IL-12/15/18-stimulated NK cells (activated NK) were co-cultured with K562 target cells (E:T ratio = 1:1) for 7 h in the presence and absence of 50 mM 2-DG. Bar graphs showing the specific percentage of cells that produce IFNγ, TNF and MIP-1β, or degranulate (CD107a), in the presence and absence of 2-DG. Specific percentage of positive cells was calculated by subtracting the percentage of positive cells for each measured function in the absence of stimuli. Means ± SEM are depicted. Statistical analyses were performed using Wilcoxon matched-pairs signed rank test. n = 7. (B) Control and activated NK cells were co-cultured with calcein-AM-labeled K562 target cells at different E:T ratios for 3 h in the presence and absence of 50 mM 2-DG. Percentage of specific lysis was measured based on calcein-AM release. Means ± SEM are depicted. Statistical analyses were performed using Wilcoxon matched-pairs signed rank test. n = 7–8. ns = non-significant, *P < 0.05, **P < 0.01.

### Increased mitochondrial activity is not sustained for long periods

OXPHOS activity tended to increase following IL-12/15/18-stimulation, but it returned to basal levels after a week in the absence of strong stimuli (Fig. 2C). We decided to further explore the mitochondrial activity by analyzing the spare respiratory capacity (SRC), defined as the difference between the maximal and basal respiration (Supplementary Fig. 5). In accordance with OXPHOS levels, activated NK cells showed increased SRC at day 0, but no significant differences were found between control and CIML NK cells at day 7 (Fig. 3A and 3B). Similarly, levels of ATP-linked respiration and non-mitochondrial oxygen consumption were significantly higher in activated NK cells at day 0, but similar between control and CIML NK cells at day 7 (Supplementary Fig. 6). To examine if the differences in the mitochondrial activity were due to changes in the cellular mitochondrial content, we next analyzed mitochondrial mass using MitoTracker staining. In contrast to other publications where 18 h of IL-2/12-stimulation increased mitochondrial mass^40^, we did not find differences in the mitochondrial mass between control and activated NK cells at day 0 (Fig. 3C, left panel). These results are in line with recent data published by Surace et al., showing that IL-12/15/18-stimulation does not induce an increment in mitochondrial mass^46^. Surprisingly, we found significant differences between control and CIML NK cells after 7 days of culture (Fig. 3C, right panel). We dismissed the hypothesis that increased mitochondrial mass was due to a higher cellular size since we found that activated and CIML NK cells were larger than control NK cells at day 0 and day 7, respectively (Supplementary Fig. 7A). These data suggest that IL-12/15/18-stimulation does not rapidly induce mitochondrial biogenesis of NK cells. Instead, mitochondrial mass is progressively increased during the following seven days of culture (Supplementary Fig. 7B). Also, our data demonstrate that increased mitochondrial mass does not necessarily correlate with augmented OXPHOS activity, since CIML NK cells show higher mitochondrial mass than control NK cells at day 7 (Fig. 3C, right panel) but similar OXPHOS and SRC rates (Fig. 2C and 3B).

**Figure 5:**
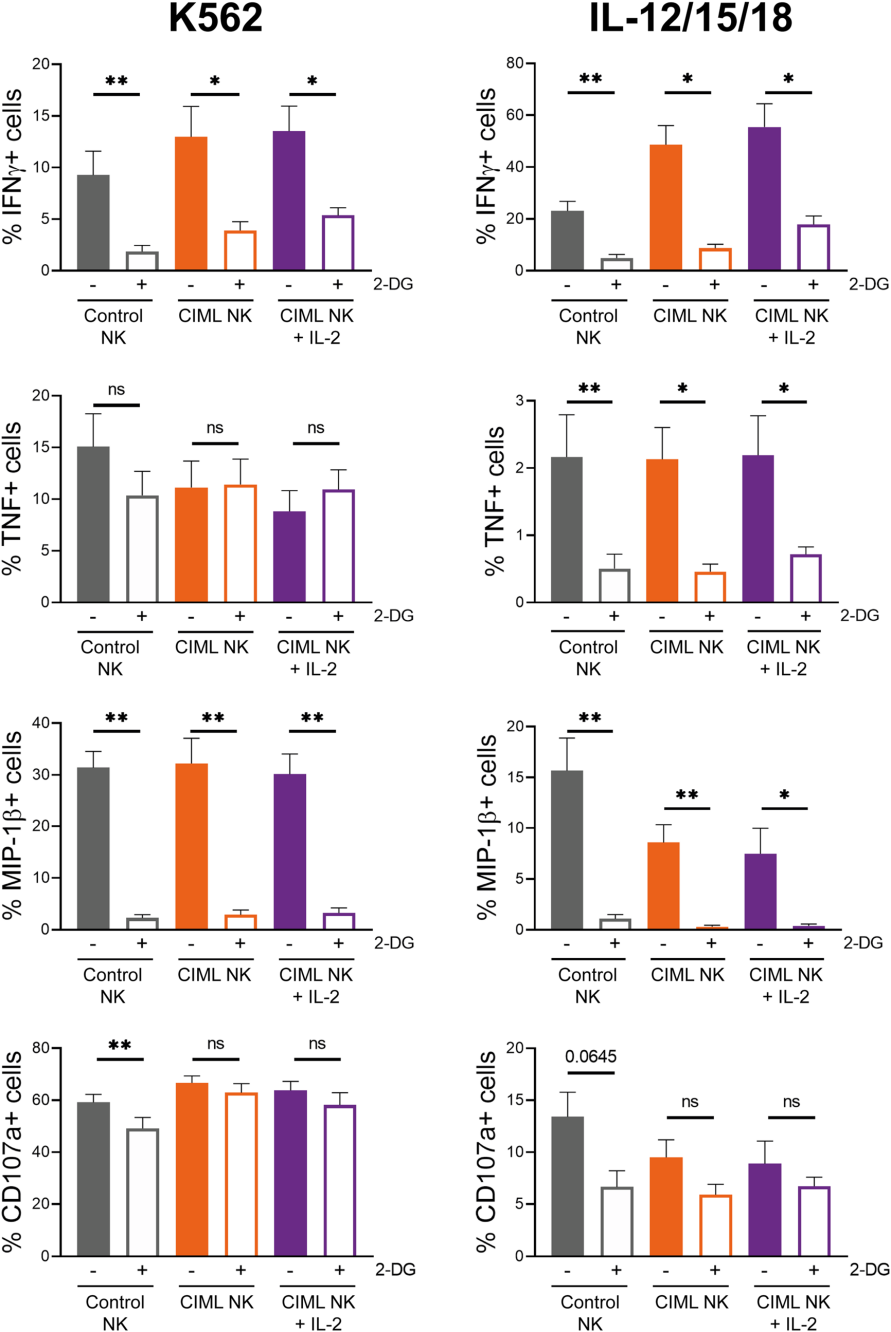
2-DG-induced inhibition of specific cytokine/chemokine production and degranulation of human cytokine-induced memory-like (CIML) NK cells at day 7. Control and activated NK cells cultured for seven days with IL-15 (control NK or CIML NK) or IL-2 (CIML NK + IL-2), were restimulated by co-culturing them with K562 target cells (E:T ratio = 1:1) or restimulated with IL-12, IL-15 and IL-18 (10, 100 and 50 ng/mL, respectively) for 7 h in the presence and absence of 50 mM 2-DG. Bar graphs showing the specific percentage of cells that produce IFNγ, TNF and MIP-1β, or degranulate (CD107a), in the presence and absence of 2-DG. Specific percentage of positive cells was calculated by subtracting the percentage of positive cells for each measured function in the absence of stimuli. Means ± SEM are depicted. Statistical analyses were performed using Wilcoxon matched-pairs signed rank test. n = 7–9. ns = non-significant, *P < 0.05, **P < 0.01.

**Figure 6:**
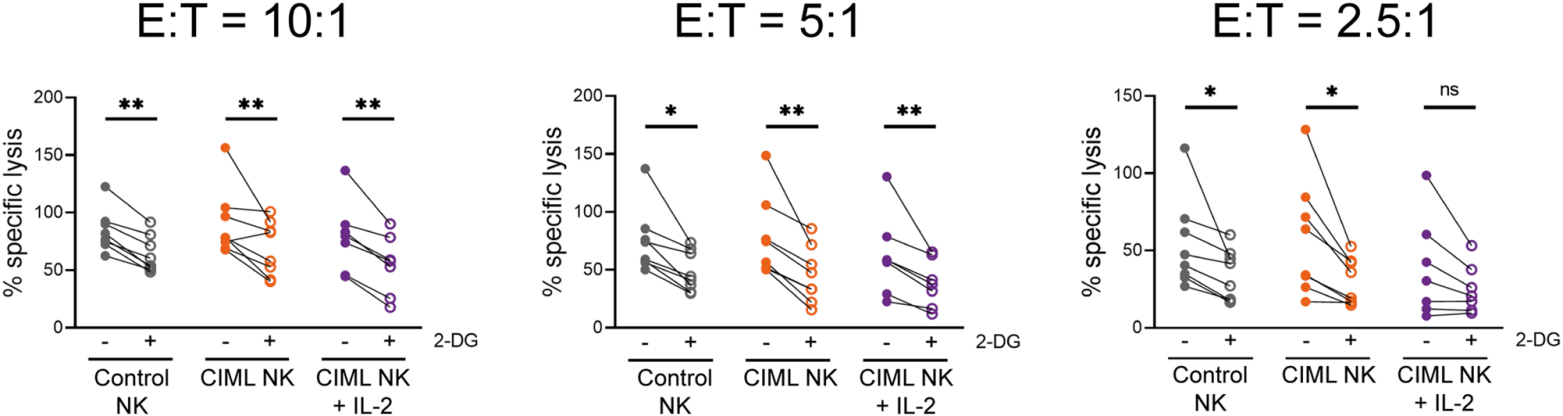
2-DG-induced inhibition of cytotoxic activity of human cytokine-induced memory-like (CIML) NK cells at day 7. Control and activated NK cells cultured for seven days with IL-15 (control NK or CIML NK) or IL-2 (CIML NK + IL-2), were co-cultured with calcein-AM-labeled K562 target cells at different E:T ratios for 3 h in the presence and absence of 50 mM 2-DG. Percentage of specific lysis was measured based on calcein-AM release. Means ± SEM are depicted. Statistical analyses were performed using Wilcoxon matched-pairs signed rank test. n = 7–8. ns = non-significant, *P < 0.05, **P < 0.01.

### 2-DG treatment differently affects distinct NK cell effector functions and is stimuli-dependent

Elevated rates of glycolysis are required for maximal IFNγ production in mouse NK cells^35, 45^. Similarly, inhibiting the glycolytic pathway by using galactose as an alternative carbon fuel decreased IFNγ production of human IL-12/15-stimulated CD56^bright^ NK cells, but not of IL-2-stimulated NK cells^32^. Interestingly, some authors have reported that inhibiting glycolysis had minimal inhibitory effects on cytokine-stimulated NK cell degranulation^32, 34^, suggesting that sensitivity to glycolytic inhibition may be different among distinct effector functions, but that also depends on the stimuli. Considering that IL-12/15/18-stimulated NK cells have elevated rates of glycolysis, we decided to explore the effect of the glycolysis inhibitor 2-DG on their different effector functions using distinct stimuli. Once 2-DG is taken up into cells, it is phosphorylated by hexokinase to 2-deoxy-D-glucose-6-phosphate, which cannot be further metabolized, resulting in its cellular accumulation and thus inducing competitive and non-competitive inhibition of glucose-6-phosphate isomerase and hexokinase, respectively^49^. Hence, activated NK cells were co-incubated with K562 target cells for 7 h in the presence and absence of 2-DG. Cytokine and chemokine production (IFNγ, TNF and MIP-1β) and degranulation (CD107a) were measured, and the percentage of specific responding cells was calculated by subtracting the percentage of positive cells, for each studied function, in the absence of stimulus. This calculation was especially relevant after the 16–18 h of cytokine-stimulation, which increased cytokine and chemokine production, and degranulation, of activated NK cells (Supplementary Fig. 8). Results showed that all the studied effector functions of both, control and activated NK cells, were reduced in the presence of 2-DG. However, IFNγ and MIP-1β production was drastically reduced, while TNF production and degranulation were only partially inhibited (Fig. 4A). We further studied the effect of glycolytic inhibition over NK cell effector functions by analyzing their cytotoxic activity against K562 target cells in the presence of 2-DG. Interestingly, our results showed that the specific lysis of K562 cells was reduced, but not completely inhibited. Notably, activated NK cells showed higher cytokine production, degranulation and cytotoxic activity than control NK cells, even in the presence of 2-DG (Fig. 4B, and Supplementary Fig. 8).

As previously described, we also cultured NK cells for 7 days with low concentrations of IL-15 or IL-2, and then analyzed their effector functions. In previous experiments, activated NK cells showed increased effector functions due to cytokine-stimulation. In contrast, after seven days of culture with low cytokine concentrations, control and CIML NK cells showed no differences in cytokine and chemokine production, and degranulation, in the absence of additional stimuli (Supplementary Fig. 9). Considering that CIML NK cells were characterized by a higher IFNγ production after cytokine-restimulation^21^, we decided to restimulate CIML NK cells either with K562 target cells or with IL-12, IL-15 and IL-18. Our results showed that IFNγ production was inhibited by 2-DG in both control and CIML NK cells, although there was a higher percentage of responding cells in the latter group, even in the presence of 2-DG (Fig. 5, and Supplementary Fig. 9). MIP-1β production was again strongly inhibited in both control and CIML NK cells (Fig. 5). Interestingly, TNF production was inhibited when NK cells were stimulated with ILs, but not when they were co-cultured with K562 target cells (Fig. 5). Of note, it should be considered that TNF production in response to cytokine-stimulation was very low in comparison with K562-stimulation. Similarly, degranulation of NK cells tended to be more inhibited by 2-DG when cells were stimulated with ILs, in comparison with K562-stimulation, although no significant differences were observed (Fig. 5). Moreover, we also analyzed cytotoxic activity of control and CIML NK cells against K562 cells at day 7. We found that their cytotoxicity was reduced but not completely inhibited by 2-DG in all the studied E:T ratios (Fig. 6).

Altogether, our results showed that effector functions of NK cells were differently affected by 2-DG. Our data suggest that IFNγ and MIP-1β production are very sensitive to glycolysis inhibition, while TNF production, degranulation and cytotoxic activity are reduced but not entirely inhibited when glycolysis is blocked. Moreover, our data suggests that 2-DG-mediated glycolysis inhibition could be dependent on the stimuli, i.e. target cells vs. cytokine-stimulation.

## Discussion

IL-12/15/18-preactivated NK cells have emerged as a powerful tool to treat leukemia^12, 19^, and their potential is also being explored in therapies against other malignancies^14–16, 18^. One of the characteristics of CIML NK cells that exemplify an advantage in cancer treatment is their persistence in the host. Ni et al. demonstrated that IL-12/15/18-preactivated NK cells were detectable 3 months after adoptive transfer in RMA-S tumor-bearing mice^13^. The long-term benefits of CIML NK cells have been proven in a mouse model of multiple myeloma, in which IL-12/15/18-stimulated NK cells were able to reduce tumor burden in bone marrow after 7 days, while the treatment with IL-15-stimulated NK cells failed^16^. Similarly, in a xenogeneic mouse model of ovarian cancer, tumor suppression was maintained after 20 days only in mice treated with CIML NK cells, but not with IL-15-stimulated NK cells^14^. Considering the long-term effects of these cells, we decided to analyze the metabolic features of IL-12/15/18-stimulated NK cells both before and after a culture period of 7 days. Current therapeutic approaches include the in vivo administration of IL-15, IL-2 or modified versions of these cytokines after cell infusion to promote survival and expansion of CIML NK cells in the host (NCT01898793, NCT02782546, NCT04024761, NCT04290546, NCT04354025, and NCT04634435, from clinicaltrials.gov). In our experimental settings, we sought to induce NK cell survival while avoiding excessive stimulation, so we cultured both control and activated NK cells with very low doses of IL-15 for one week, as others have previously done^12, 26^. Additionally, we tried to mimic the conditions in which infused CIML NK cells in clinical trials are helped to expand^12^, by culturing them with IL-2. Although we used low doses of IL-2 (20 IU/mL), we have previously described that this concentration is enough to promote proliferation of IL-12/15/18-preactivated NK cells^22^. Therefore, our experimental design allowed us to in vitro study the metabolism and effector functions of resting and proliferating CIML NK cells, in a somehow comparable manner as it may be happening after their administration in patients.

We first decided to analyze the expression of nutrient transporters. Our data is in accordance with results published by others, confirming that the expression of nutrient transporters in NK cells can be upregulated in response to different stimuli^32, 35, 39–46, 50^. Interestingly, our results showed that 7 days after cytokine-preactivation, CIML NK cells retain increased expression of CD98 and GLUT1, although the relevance of these transporters in the tumor context is still unknown. Inhibition of CD98 activity with D-phenylalanine has been found to completely abolish NKG2D-induced IFNγ production and degranulation of NK cells^44^. Similarly, inhibition of amino acid transport through CD98/LAT1 complex with 2-aminobicyclo-(2,2,1)-heptane-2-carboxylic acid (BCH) inhibited IFNγ and granzyme B production of IL-2/18- and IL-2/12-stimulated NK cells^40, 41^. Overall, these reports showed that blocking CD98 activity limits NK cells effector functions, but to our knowledge, an elevated expression of CD98 has not been linked to improved functionality. Nonetheless, it may represent an advantage in an amino acid-depleted environment such as the tumor microenvironment (TME), where there is a competition for nutrients between tumor cells, myeloid-derived suppressor cells and lymphocytes^38, 51^. Likewise, experiments in which glucose has been substituted with galactose revealed that glycolytic rates and effector functions of IL-2/12- or IL-12/15-stimulated NK cells decreased^32, 35^, thus highlighting the relevance of glucose availability. Indeed, in a mouse model, NK cells from lung cancer microenvironment showed lower levels of glycolysis and reduced effector functions^37^. Moreover, the authors found that inhibition of FBP1 partially restored glycolytic activity and effector functions of lung NK cells^37^. Although there are multiple factors that could modulate glycolysis and functionality of NK cells in the TME^38^, an increased GLUT1 expression may mitigate the tumor-induced decrease of glycolytic activity due to a higher glucose uptake and thus be beneficial for NK cell functions. Therefore, a higher CD98 and GLUT1 expression could be helpful to support the functionality of CIML NK cells in nutrient-restricted environments. Of note, some of the metabolic changes appreciated in IL-12/15/18-stimulated NK cells tend to be more exacerbated when cultured with IL-2, suggesting that IL-2 may support some of the metabolic changes of CIML NK cells. Also, it is interesting to note that different NK cell subsets showed different expression of nutrient transporters. We found differences between CD56^bright^ and CD56^dim^ NK cell subsets at day 0, but CD56 expression is modulated during the following culture period, so these subsets could not be identified at day 7. Different expression of nutrient transporters has been also reported in other subsets. For instance, KIR-educated NK cells showed increased GLUT1 expression compared to NKG2A-educated NK cells^52^. It would be worth to explore whether these educated NK cell subsets respond also differently to IL-12/15/18-stimulation, and to evaluate if the metabolic changes are maintained when cells return to a resting state.

Current knowledge indicates that there are several differences between non-preactivated and CIML NK cells at phenotypic and functional levels^12–17, 21–25, 27–29, 53, 54^. Considering that metabolism supports cellular functions, we hypothesized that CIML NK cells may also differ in their metabolic profile. Others have previously reported that NK cell stimulation induced an increase in the glycolytic activity^32, 34, 35, 39, 41, 45, 50, 55^. In accordance, our results showed that NK cells have a higher glycolysis levels following IL-12/15/18-stimulation for 16–18 h. Of note, even though 2-NBDG assay has been widely used to measure glucose uptake, recent findings suggested that this assay should be carefully interpreted^56^. Remarkably, we found that the increase in the glycolytic machinery is not a transient event, and that CIML NK cells retain a metabolic profile shifted towards glycolysis for at least one week after IL-12/15/18-preactivation. It would be interesting to study if other stimuli could also induce a sustained metabolic switch. For instance, it has been reported that adaptive NK cells from patients infected with cytomegalovirus (CMV) showed higher glycolytic metabolism than canonical NK cells^57^, suggesting that viral infection could induce this glycolytic profile. Nonetheless, adaptive and CIML NK cells differ in other metabolic features. Adaptive NK cells from CMV + individuals showed increased OXPHOS activity and higher SRC than canonical NK cells^57^. IL-12/15/18-stimulated NK cells share the increment in OXPHOS and SRC rates, but these parameters return to basal levels after seven days. Nevertheless, in our experimental settings, CIML NK cells are not exposed to activating stimuli (e.g. viral infected cells) during the culture period of seven days, which may explain why OXPHOS activity returns to basal levels. Furthermore, mitochondrial mass of mouse NK cells did not show significant changes one week after CMV infection, and then decreased during the following days in the contraction-to-memory phase^58^. Contrary to adaptive NK cells, CIML NK cells showed a progressive increase in the mitochondrial mass. Hence, adaptive and CIML NK cells exhibit different metabolic changes. A recent report revealed that, following IL-12/15/18-stimulation, mitochondrial membrane potential is increased in CD56^bright^ but decreased in CD56^dim^ NK cells^46^. It would be possible that CIML NK cells accumulate dysfunctional mitochondria during the resting period that led to differences in mitochondrial mass found at day 7. Of note, CMV + and CMV- donors were not segregated in our experiments, but it would be worth to explore if CMV status could have an effect in the response to IL-12/15/18-stimulation, and if different metabolic changes are induced in adaptive or canonical NK cells. Nonetheless, considering the differences in the mitochondrial content of control and CIML NK cells, it would be interesting to study in future works if CIML NK cells are prone to increase more rapidly OXPHOS activity than canonical NK cells when restimulated after a resting period, or if the superior mitochondrial mass belongs to dysfunctional mitochondria.

Our experiments were performed in a system with optimal conditions for NK cells. However, in the tumor context NK cells have to face a suppressive microenvironment that negatively affects to both metabolism and effector functions^38^. In a recent report, control and CIML NK cells were cultured for seven days with ascites supernatant from patients with ovarian cancer, and then their effector functions were measured by co-culturing with ovarian cancer cell lines. Interestingly, the authors found that IL-12/15/18-preactivated NK cells showed a higher IFNγ production than control NK cells, suggesting that CIML NK cells may have the ability to overcome the suppressive soluble microenvironment^14^. To further explore this idea, we decided to study effector functions of CIML NK cells in metabolically restricting conditions. Considering that NK cells showed reduced glycolytic activity in the TME^37^ and to assess the relevance of this metabolic pathway for NK cell functions, we stimulated NK cells with either target cells or cytokines in the presence of the glycolysis inhibitor 2-DG. Previous works have shown that low doses (1 mM) of this metabolic inhibitor reduced IFNγ and/or granzyme B production of murine NK cells in vitro following stimulation with IL-15, IL-2/12 or anti-NK1.1 plus IL-2^34, 35, 45^. Remarkably, in vivo administration of 2-DG reduced IFNγ production but not TNF production of NK cells following poly(I:C)-mediated activation^35^. Human NK cells are more resistant to 2-DG than murine NK cells^34^, so direct comparisons should be carefully considered. Nonetheless, our data confirmed that distinct effector functions of human NK cells also have different sensitivity to 2-DG-induced glycolytic inhibition. Also, it would be interesting to explore if these differential effects could be due to off-target effects of 2-DG.

In agreement with the initial definition of CIML NK cells^21^, increased production of IFNγ was detected when CIML NK cells were restimulated with either IL-12/15/18 or K562 cells. However, these differences were not detected in the rest of the studied parameters. Interestingly, our results revealed that CIML NK cells restimulated with IL-12/15/18 at day 7 showed reduced TNF production and degranulation in the presence of 2-DG. Contrarily, those effector functions were not affected by 2-DG when NK cells were co-cultured with the K562 tumor cell line. It may be possible that K562 target cells hijacked 2-DG from media and thus NK cells were less exposed to this inhibitor. However, we have excluded this possibility because IFNγ and MIP-1β production were severely inhibited when 2-DG was added, so NK cells are also presumably exposed to this inhibitor. It has been previously described that IFNγ production is reduced by the combination of 2-DG and other metabolic inhibitors in murine NK cells stimulated through NK1.1, but no negative effects were found following IL-12/18 stimulation^59^. Therefore, our results support the idea that metabolic requirements for a specific function could be different depending on the stimuli.

The effect of 2-DG on NK cell cytotoxic activity is still unclear. NK cells from CMV-infected mice receiving 2-DG treatment showed reduced target clearance^34^. The same authors described that human NK cell cytotoxic activity against K562 cells was reduced when IL-15-stimulated NK cells were exposed to 2-DG for 24 h prior to co-culturing NK and K562 cells^34^. Contrarily, NK cells pretreated with 2-DG for 4 h prior to the co-culture with K562 cells did not show defects in the killing of target cells, both in the presence and absence of 2-DG during the co-culture^55^. Similar results were obtained by pretreating expanded human NK cells with 2-DG for 3 h and co-culturing them with 721.221 target cells^60^. However, the authors pointed that 2-DG-induced effect may be reversible, so its potential inhibition of NK cell-mediated cytotoxicity could be lost when the inhibitor is washed out^60^. We assessed this issue by adding 2-DG during the co-culture period and we found that NK cell cytotoxicity against K562 cells is reduced by 2-DG. Discrepancies with previous studies could be explained because of the different experimental settings, such as co-culture duration, 2-DG concentration and different E:T ratios, among others. Importantly, it is known that NK cell target recognition involves an array of receptors that may be different depending on the nature of the target cell. This fact very possibly will have a relevant effect on the metabolic requirements of the effector functions of human NK cells, since, as we have shown, the requirements are stimuli-dependent. Moreover, it should be considered that in our experimental system K562 cells could also be affected by 2-DG, and that different target cells may be more or less dependent on glycolytic metabolism and thus have a higher or lower sensitivity to 2-DG. Therefore, the presence of 2-DG may differently affect to NK cell cytotoxicity against other cell lines, such as 721.221 cells (data not shown). It has been reported that 2-DG interferes with protein N-glycosylation process, which prevented the expression of NKG2D ligands in multiple cell lines^61, 62^. This fact adds an additional layer of complexity to understand the specific effect of 2-DG over CIML NK cell cytotoxicity against different target cells. Remarkably, our results showed that, at day 0, activated NK cells showed higher cytotoxic activity than control NK cells even in the presence of 2-DG. These results suggest that CIML NK cells could maintain higher antitumor activity in glycolysis-restricting conditions, such as the TME^38^. Nevertheless, it would be very important to further explore whether CIML NK cells could overcome metabolic suppression with limiting concentrations of glucose or by directly studying CIML NK cells in the TME, and understand the contribution of other metabolic pathways to their increased effector functions.

Immunometabolism could greatly influence the outcome of cancer immunotherapies. This idea has gained acceptance during the last years and, consequently, several strategies have been proposed to induce metabolic reprogramming of immune cells both in vivo and in vitro^63^. Our work has demonstrated that IL-12/15/18-stimulation induces some metabolic changes that are maintained when cells return to a resting state, thus revealing that human CIML NK cells show somehow durable metabolic changes. Furthermore, we have explored the different glycolytic requirements for the effector functions of NK cells. These findings would help to design future therapies in which NK cells could be metabolically reprogrammed prior to cell infusion, and highlight the necessity to continue exploring the impact of the TME over NK cell metabolism to find new means of improving NK cell-based cancer immunotherapy.

## Materials and methods

### Samples and cell culture

Blood samples (buffy coats) from adult healthy donors were collected through the Basque Biobank (https://www.biobancovasco.org). The Basque Biobank complies with the quality management, traceability and biosecurity, set out in the Spanish Law 14/2007 of Biomedical Research and in the Royal Decree 1716/2011. All subjects provided written and signed informed consent in accordance with the Declaration of Helsinki. The protocol was approved by the Basque Ethics Committee for Clinical Research (PI + INC-BIOEF 2014–02, PI + CES + INC-BIOEF 2017–03 and PI2014079). Fresh peripheral blood mononuclear cells (PBMCs) were obtained from buffy coats by Ficoll Paque Plus (GE Healthcare) density gradient centrifugation. NK cells were isolated from PBMCs by negative depletion with human NK cell Isolation Kit (Miltenyi Biotec). Cells were cultured in RPMI 1640 medium supplemented with GlutaMAX (Gibco), 10% heat-inactivated Fetal Bovine Serum (FBS) (HyClone), 1% non-essential amino acids (Gibco), 1% Sodium Pyruvate (Gibco) and 1% Penicillin–Streptomycin (Gibco). PBMCs or purified NK cells were stimulated for 16–18 h with 10 ng/mL rhIL-12 (Miltenyi Biotec), 100 ng/mL rhIL-15 (Miltenyi Biotec) and 50 ng/mL rhIL-18 (MBL International Corporation), or cultured in media alone for the same time. Cells were then washed three times and cultured with 1 ng/mL rhIL-15 or 20 IU/mL rhIL-2 (Miltenyi Biotec). After 4 days, media and cytokines (IL-15 or IL-2) were replaced, and cells were cultured for 3 more days. The time-points after the 16–18 h stimulation, and after seven days of culture, are referred to as Day 0 and Day 7, respectively (Supplementary Fig. 2). The K562 cell line was cultured in the same media than NK cells supplemented with 5 µg/mL of Plasmocin (InvivoGen) and was routinely tested for mycoplasma infection with Venor GeM Classic detection kit (Minerva Biolabs).

### Flow cytometry

Extracellular staining was performed by incubating cells for 30 min on ice with the following fluorochrome-conjugated mouse anti-human antibodies: BV421 anti-CD71 (M-A712), BV510 anti-CD3 (UCHT1), BV510 anti-CD14 (MφP9) and PE anti-CD98 (UM7F8) from BD Biosciences; and PE-Vio770 anti-CD56 (REA196) from Miltenyi Biotec. Cells were then washed with PBS containing 2.5% Bovine Serum Albumin (BSA) (Sigma-Aldrich), and, for intracellular staining, cells were fixed and permeabilized with Fixation/Permeabilization solution (BD Biosciences), following manufacturer´s protocol. Next, intracellular staining was performed by incubating cells for 30 min on ice with the following fluorochrome conjugated mouse anti-human antibodies: BV421 anti-IFNγ (B27) and FITC anti-MIP-1β (D21-1351) from BD Biosciences; and APC anti-TNF (Mab11) from BioLegend. The following fluorochrome conjugated rabbit anti-human antibodies were also used in the intracellular staining: FITC anti-GLUT3 (polyclonal) and PE anti-GLUT1 (EPR3915) from Abcam. Brilliant Stain Buffer (BD Biosciences) was used during the simultaneous incubation with BV421 and BV510 dyes. Cell viability was determined by staining cells for 30 min on ice with LIVE/DEAD Fixable Near-IR Dead Cell Stain Kit (Invitrogen) prior to extracellular staining. Mitochondrial mass was measured by incubating cells with 150 nM MitoTracker Green FM (Invitrogen) for 25 min at 37 °C after the extracellular staining. 2-NBDG uptake assay was performed by incubating cells with 50 µM 2-NBDG (2-(N-(7-Nitrobenz-2-oxa-1,3-diazol-4-yl)Amino)-2-Deoxyglucose) (Invitrogen) for 90 min at 37 °C prior to cell staining. NK cells were gated within viable lymphocytes as CD3-CD14-CD56 + cells (Supplementary Fig. 1^10^. Flow cytometry was performed with MACSQuant Analyzer 10 (Miltenyi Biotec). Data was analyzed with FlowLogic v.7.3 software.

### Extracellular flux assay

Purified NK cells were washed and resuspended in Seahorse XF DMEM Medium (pH 7.4, Agilent Technologies) supplemented with 10 mM glucose, 2 mM glutamine and 1 mM pyruvate. 96 well microplates were treated with Cell-Tak (Corning) and cells were plated into six replicates at 200,000 cells/well. Extracellular acidification rate (ECAR) and oxygen consumption rate (OCR) were determined using a Seahorse XFe96 Extracellular Flux Analyzer (Agilent Technologies). Seahorse XF Cell Mito Stress Test (Agilent Technologies) was used following manufacturer’s indications, using 1, 1 and 0.5 µM of oligomycin, FCCP and rotenone/antimycin A, respectively. Data was analyzed with Seahorse Wave Desktop software v.2.6 (Agilent Technologies).

### Functional assay

NK cells were washed and plated at 200,000 cell/well (10^6^ cell/mL) in 96 U-bottom well plates in the presence and absence of 50 mM 2-Deoxy-D-glucose (2-DG) (Sigma-Aldrich). To stimulate them, K562 target cells at 1:1 effector:target (E:T) ratio, or IL-12, IL-15 and IL-18 (10, 100 and 50 ng/mL, respectively) were added. Mouse anti-human PE anti-CD107a mAb (REA792, from Miltenyi Biotec) was added and cells were cultured at 37 °C for one hour. Then, GolgiPlug and GolgiStop (brefeldin A and monensin, respectively) protein transport inhibitors (BD Biosciences) were added, following manufacturer’s recommendations, and cells were cultured for additional 6 h at 37 °C. Plates were pulse centrifuged at 200 g for 1 min prior to each incubation period. Cells were then collected, stored overnight at 4 °C, and stained and analyzed by flow cytometry the next day. The percentage of specific response was calculated as the percentage of stimulated cells that are positive for a specific function minus the percentage of non-stimulated cells that are positive for the same function.

### Cytotoxicity assay

The assay was performed following a previously described protocol^22^. Briefly, K562 target cells were incubated for 30 min at 37 °C in the presence of 15 µM calcein-AM (Invitrogen). PBMCs were washed and plated into three replicates in 96 U-bottom well plates with labeled target cells at different E:T ratios, with 5,000 target cells per well. Plates were pulse centrifuged at 200 g for 1 min and then cultured for 3 h at 37 °C in the presence and absence of 50 mM 2-DG. Then, plates were centrifuged and 75 µL of the supernatant were transferred to black 96 well plates to measure calcein-AM release with a Varioskan Flash fluorimeter (Thermo Fisher Scientific), with a configuration of excitation/emission of 485/518 nm. Mean of the triplicates of each condition was calculated for the analysis. Medium fluorescence was calculated without cells, in the presence and absence of 2-DG. Spontaneous release was measured by culturing target cells without PBMCs, in the presence and absence of 2-DG. Maximum release was measured by culturing labeled target cells with 2% Triton X-100 (Sigma-Aldrich), in the presence and absence of 2-DG. Triton fluorescence was measured with media plus 2% Triton X-100 without cells, in the presence and absence of 2-DG. The percentage of specific lysis was calculated with the following formula:

$$\frac{{\left( {{\text{Test}} {\text{fluor}}. - {\text{Medium}} {\text{fluor}}.} \right) - \left( {{\text{Spont}}.{\text{ release}} - {\text{Medium}} {\text{fluor}}.} \right)}}{{\left( {{\text{Max}}.{\text{ release}} - {\text{Triton}} {\text{fluor}}.} \right) - \left( {{\text{Spont}}.{\text{ release}} - {\text{Medium}} {\text{fluor}}.} \right)}}{ \times }100$$

### Statistical analysis and data representation

GraphPad Prism v.8.4 was used for graphical representation and statistical analysis. Data was represented showing mean ± standard error of the mean (SEM). Non-parametric Wilcoxon matched-pairs signed rank test were used to determine significant differences. ns: non-significant, *P < 0.05, **P < 0.01.

## Supplementary Information

Supplementary Information

## Data Availability

The datasets used and/or analyzed during the current study are available from the corresponding authors on reasonable request.
