# Supplementary material for: Metabolic changes of Interleukin-12/15/18-stimulated human NK cells
Source: Sci Rep. 2021 Mar 19;11:6472. doi: 10.1038/s41598-021-85960-6 (PMC7979769; doi:10.1038/s41598-021-85960-6)
Supplement: Supplementary file 1 — Supplementary Information [file 41598_2021_85960_MOESM1_ESM.pdf]

Supplementary Information for

Metabolic changes of Interleukin-12/15/18-stimulated human NK cells

Iñigo Terrén[1], Ane Orrantia[1], Alba Mosteiro[1], Joana Vitallé[1], Olatz Zenarruzabeitia[1*] & Francisco Borrego[1,2*]

[1]Immunopathology Group, Biocruces Bizkaia Health Research Institute, Barakaldo, Spain
[2]Ikerbasque, Basque Foundation for Science, Bilbao, Spain

*Correspondence:
Olatz Zenarruzabeitia, PhD
olatz.zenarruzabeitiabelaustegui@osakidetza.eus
Francisco Borrego MD, PhD
francisco.borregorabasco@osakidetza.eus

This file includes:

- Supplementary Figure 1. Expression of nutrient transporters in human IL-12/15/18-stimulated NK cells.
- Supplementary Figure 2. Schematic representation of experimental design.
- Supplementary Figure 3. Effect of low doses of IL-15 on CD71 expression at day 7.
- Supplementary Figure 4. Glycolytic activity of human IL-12/15/18-stimulated NK cells.
- Supplementary Figure 5. Mitochondrial respiration of human IL-12/15/18-stimulated NK cells.
- Supplementary Figure 6. Mitochondrial respiration-linked parameters of human IL-12/15/18-stimulated NK cells.
- Supplementary Figure 7. Evolution of mitochondrial mass and cellular size of human IL-12/15/18-stimulated NK cells.
- Supplementary Figure 8. 2-DG-induced inhibition of cytokine/chemokine production and degranulation of human IL-12/15/18-stimulated NK cells at day 0.
- Supplementary Figure 9. 2-DG-induced inhibition of cytokine/chemokine production and degranulation of human cytokine-induced memory-like (CIML) NK cells at day 7.
- Supplementary Figure 10. Gating strategy of purified NK cells.

Supplementary Figure 1

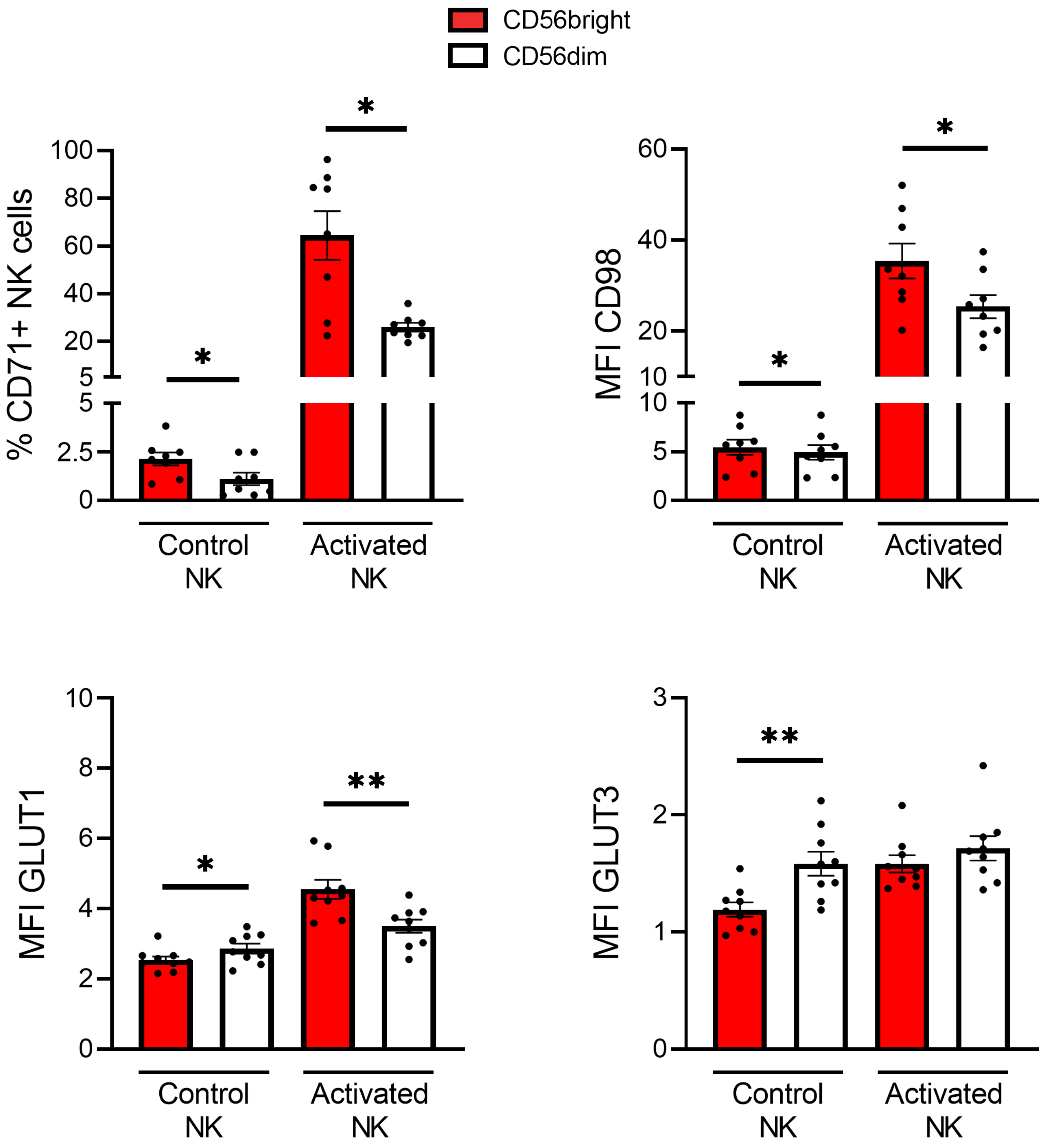

Supplementary Figure 1. Expression of nutrient transporters in human IL-12/15/18-stimulated NK cells. Bar charts representing the percentage of control NK and IL-12/15/18-stimulated (activated NK) NK cells expressing the transferrin receptor CD71, and expression levels of the heavy chain of multiple heterodimeric amino acid transporters CD98, and glucose transporters GLUT1 and GLUT3, measured as median fluorescence intensity (MFI). Means ± SEM are depicted. Statistical analyses were performed using Wilcoxon matched-pairs signed rank test. Each dot represents an independent experiment from a different donor (n = 8-9). *p<0.05, **p<0.01.

Supplementary Figure 2

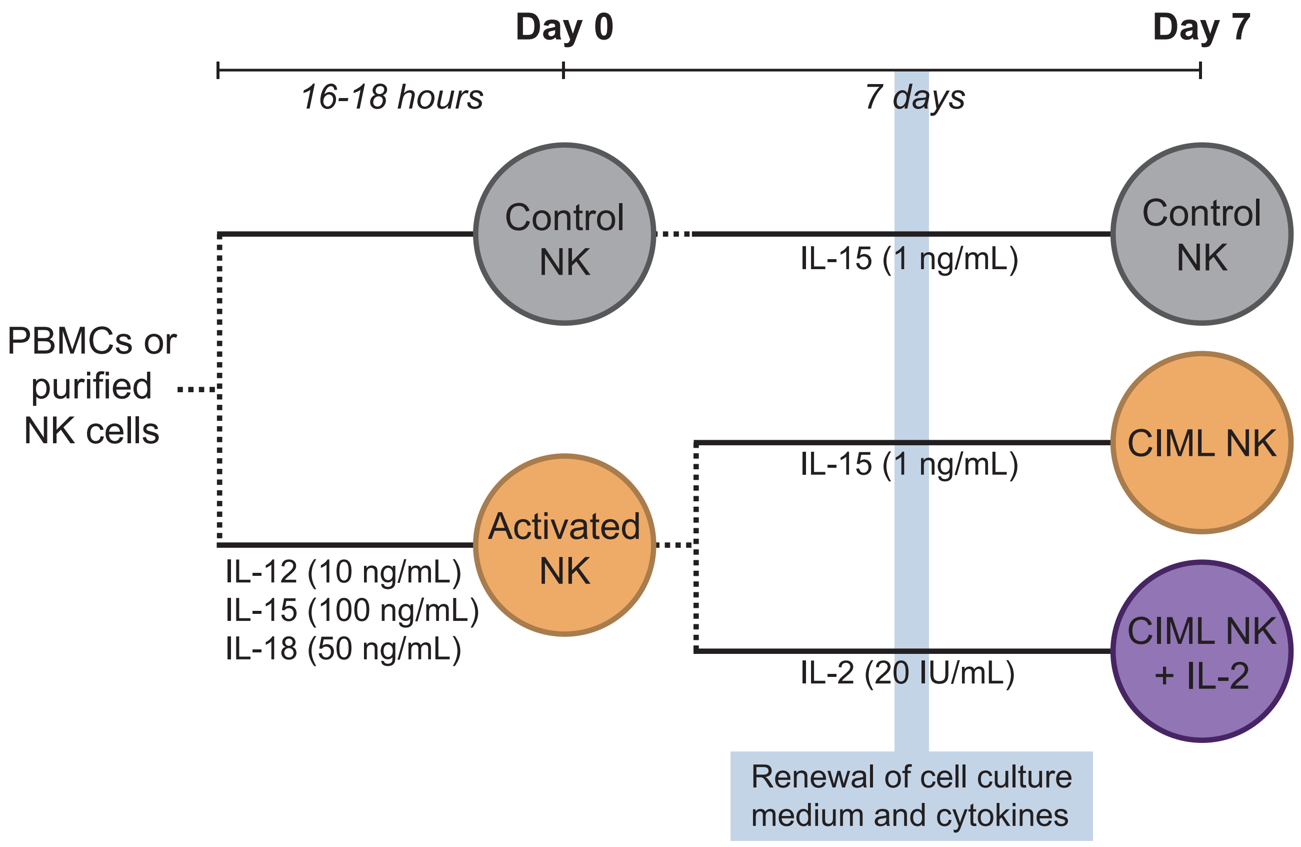

Supplementary Figure 2. Schematic representation of experimental design. PBMCs or purified NK cells (depending on the assay, as indicated in the methodology section) were cultured for 16-18 hours with media alone (control NK) or with a mixture of IL-12, IL-15 and IL-18 (10, 100 and 50 ng/mL, respectively) (activated NK). At this time point (Day 0), cells were collected, washed and analyzed. Additionally, cells were further cultured for seven days in media containing 1 ng/mL IL-15 or 20 IU/mL IL-2. Media and cytokines were renewed four days after the Day 0. After seven days (Day 7), cells were collected, washed and analyzed.

Supplementary Figure 3

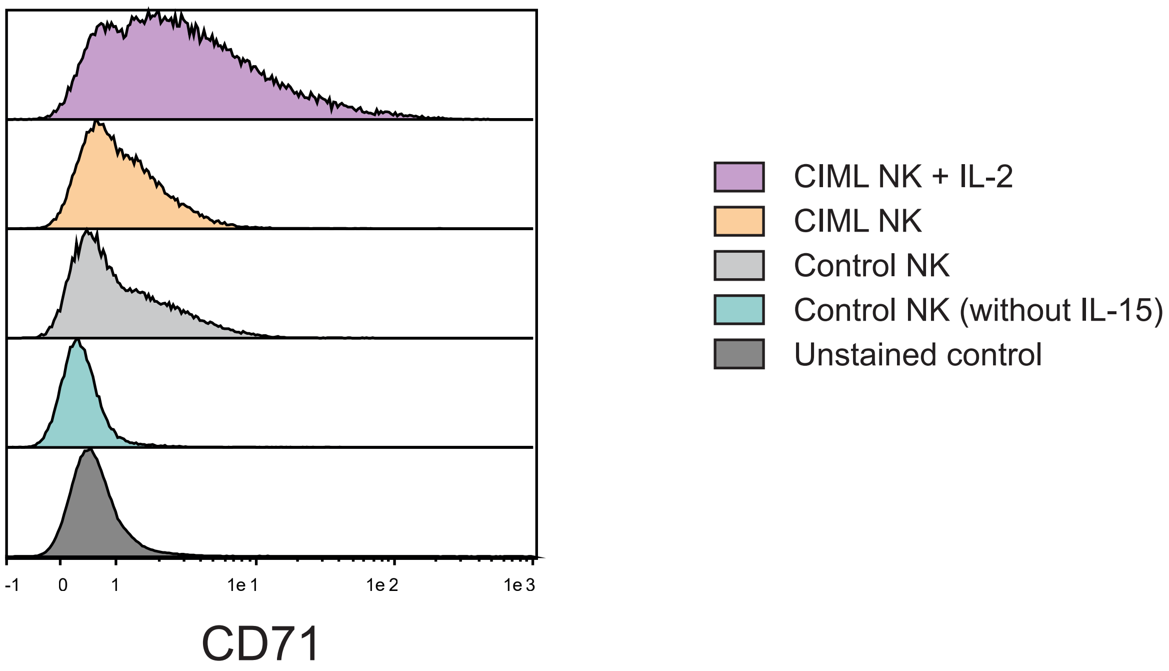

Supplementary Figure 3. Effect of low doses of IL-15 on CD71 expression at day 7. Control NK and activated NK cells were cultured in media without cytokines, or with IL-15 (1 ng/mL) or IL-2 (20 IU/mL) for seven days. Media was replaced during this culture period, as explained in the methodology section. Histograms show the expression of transferrin receptor CD71, measured within viable NK cells.

Supplementary Figure 4

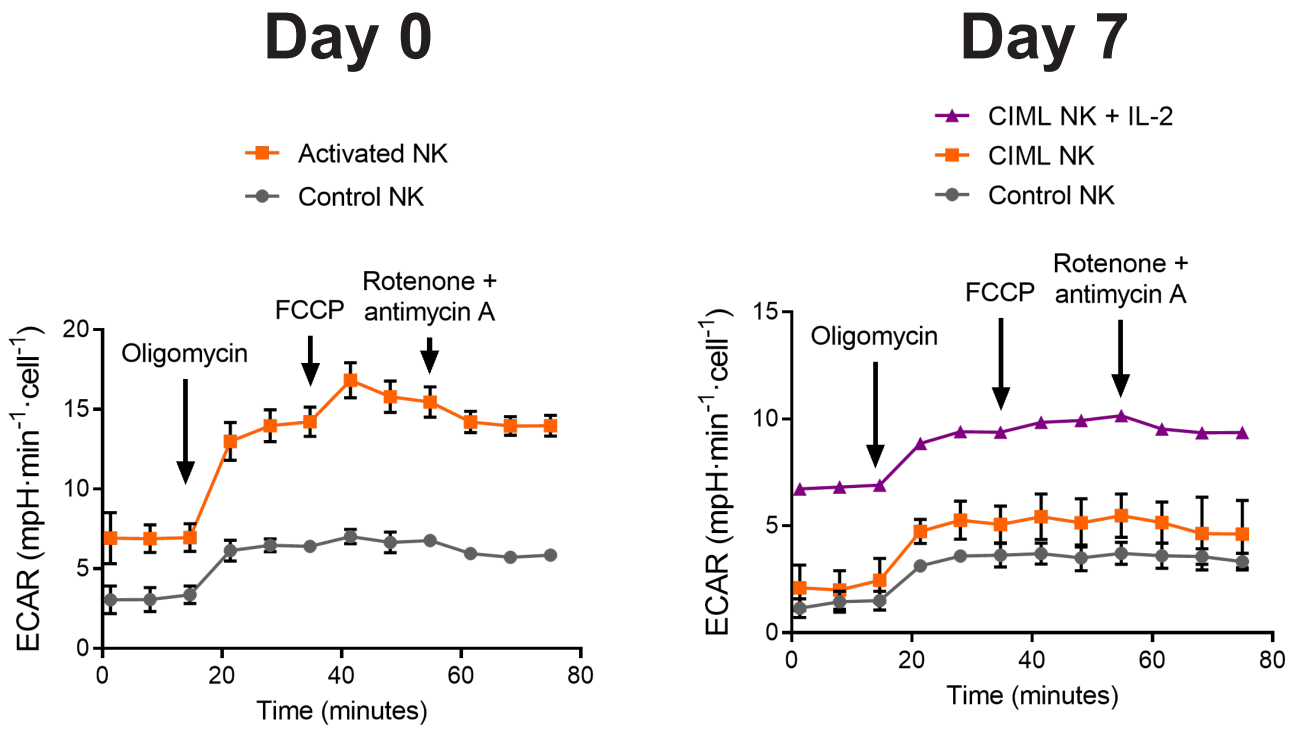

Supplementary Figure 4. Glycolytic activity of human IL-12/15/18-stimulated NK cells. ECAR values from a representative experiment of Seahorse XF analyzer with Mito Stress kit. Means ± standar deviation are depicted.

Supplementary Figure 5

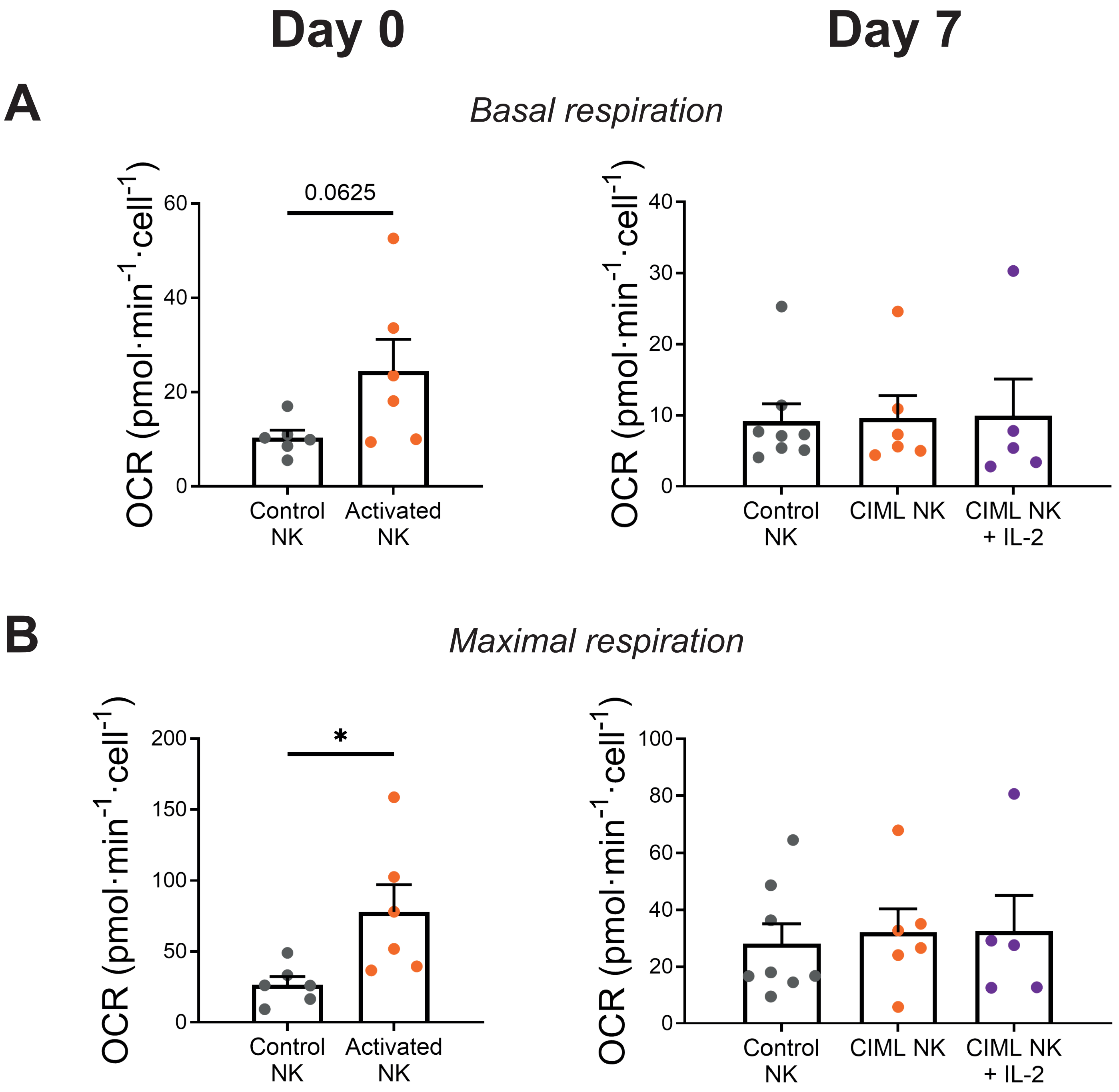

Supplementary Figure 5. Mitochondrial respiration of human IL-12/15/18-stimulated NK cells. Bar charts representing (A) basal respiration and (B) maximal respiration (i.e. respiration levels following addition of FCCP) rates, measured as oxygen consumption rate (OCR). Graphs show data of control NK cells and IL-12/15/18-stimulated NK cells (activated NK) (Day 0, left column), and after 7 days of culture with IL-15 (control NK or CIML NK) or IL-2 (CIML NK+IL-2) (Day 7, right column). Means ± SEM are depicted. Statistical analyses were performed using Wilcoxon matched-pairs signed rank test. Each dot represents an independent experiment from a different donor (n = 5-8). *p<0.05.

Supplementary Figure 6

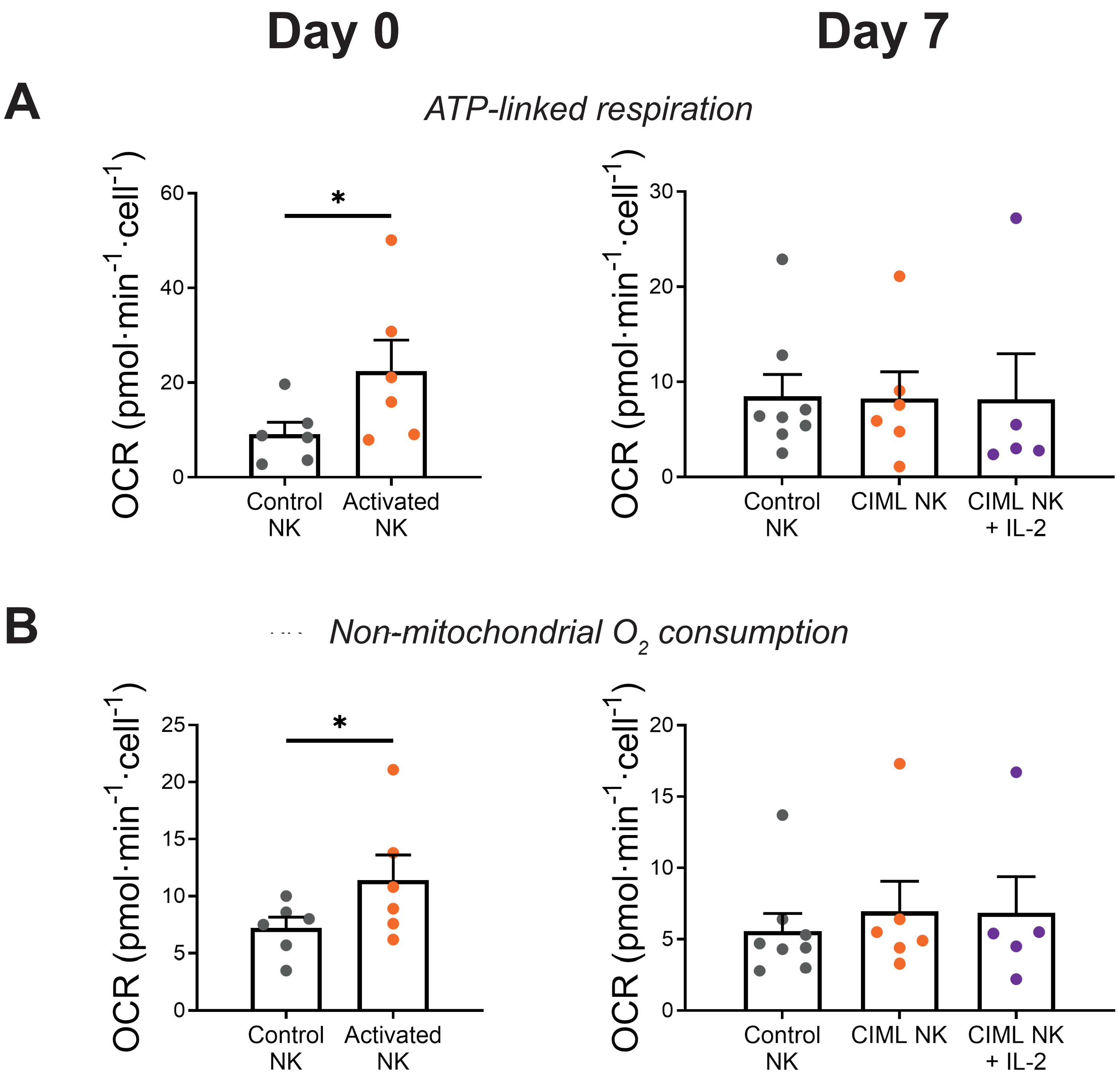

Supplementary Figure 6. Mitochondrial respiration-linked parameters of human IL-12/15/18-stimulated NK cells. Bar charts representing (A) ATP-linked respiration, measured by the decrease in oxygen consumption rate (OCR) when cells are exposed to ATP synthase inhibitor oligomycin, and (B) non-mitochondrial oxygen consumption, measured as the OCR of cells exposed to oligomycin, FCCP, rotenone and antimycin A. Graphs show data of control NK cells and IL-12/15/18-stimulated NK cells (activated NK) (Day 0, left column), and after 7 days of culture with IL-15 (control NK or CIML NK) or IL-2 (CIML NK+IL-2) (Day 7, right column). Means ± SEM are depicted. Statistical analyses were performed using Wilcoxon matched-pairs signed rank test. Each dot represents an independent experiment from a different donor (n = 5-8). *p<0.05.

Supplementary Figure 7

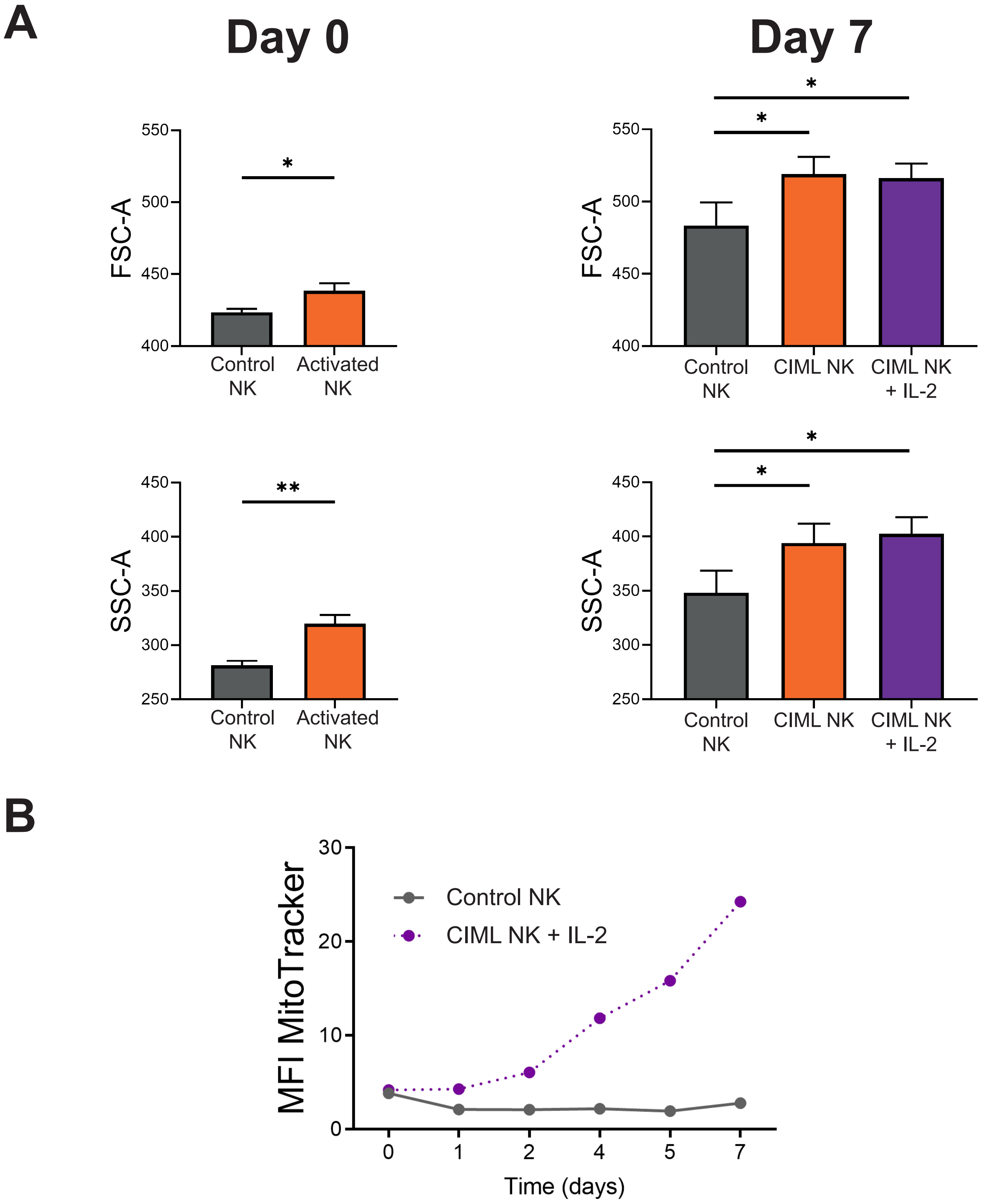

Supplementary Figure 7. Evolution of mitochondrial mass and cellular size of human IL-12/15/18-stimulated NK cells. (A) Bar graphs representing cellular size and granularity, measured by forward (FSC) and side scatter (SSC) values, of control NK cells and IL-12/15/18-stimulated NK cells (activated NK) (Day 0, left column), and after 7 days of culture with IL-15 (control NK or CIML NK) or IL-2 (CIML NK+IL-2) (Day 7, right column). Means ± SEM are depicted. Statistical analyses were performed using Wilcoxon matched-pairs signed rank test (n = 8-9). *$p<0.05$, **$p<0.01$. (B) Repeated measures of mitochondrial mass during the culture period of seven days, measured as the median fluorescence intensity (MFI) of MitoTracker Green (n = 1).

Supplementary Figure 8

A

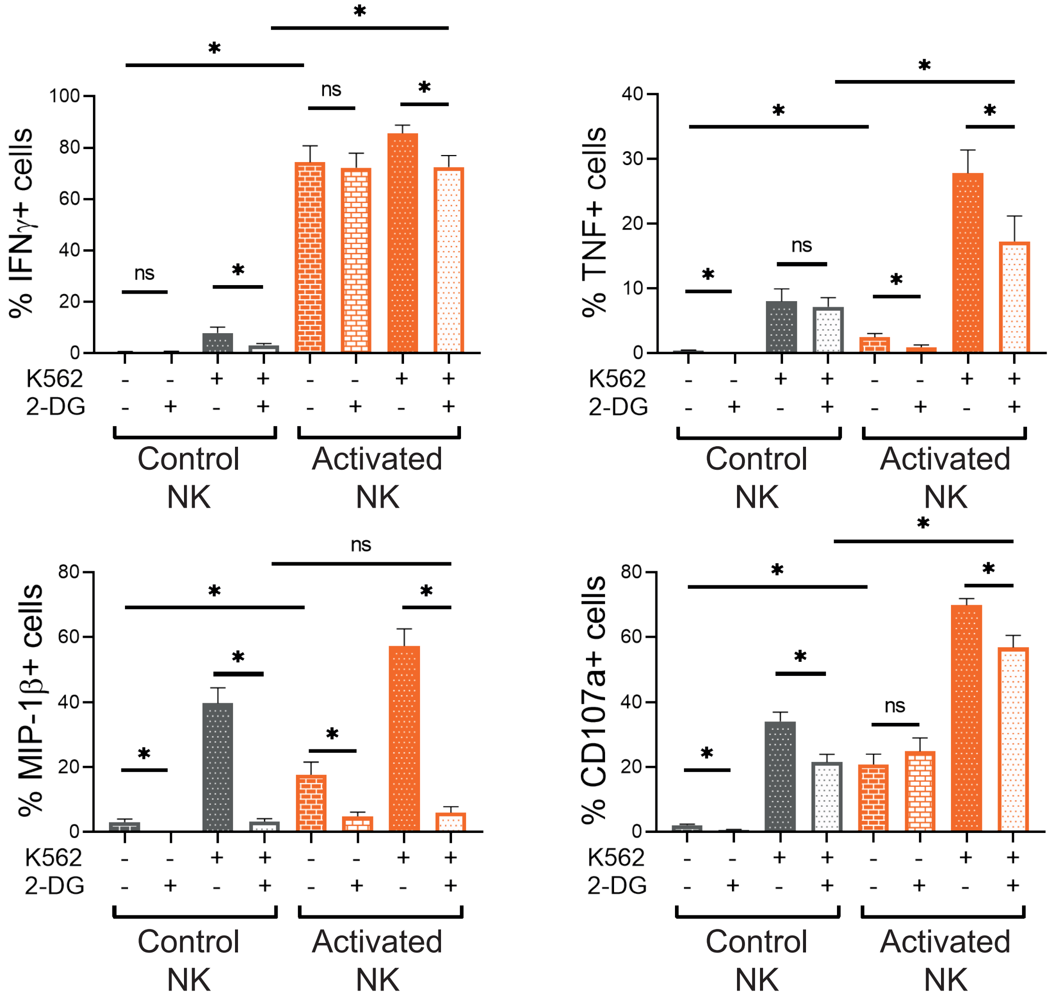

B

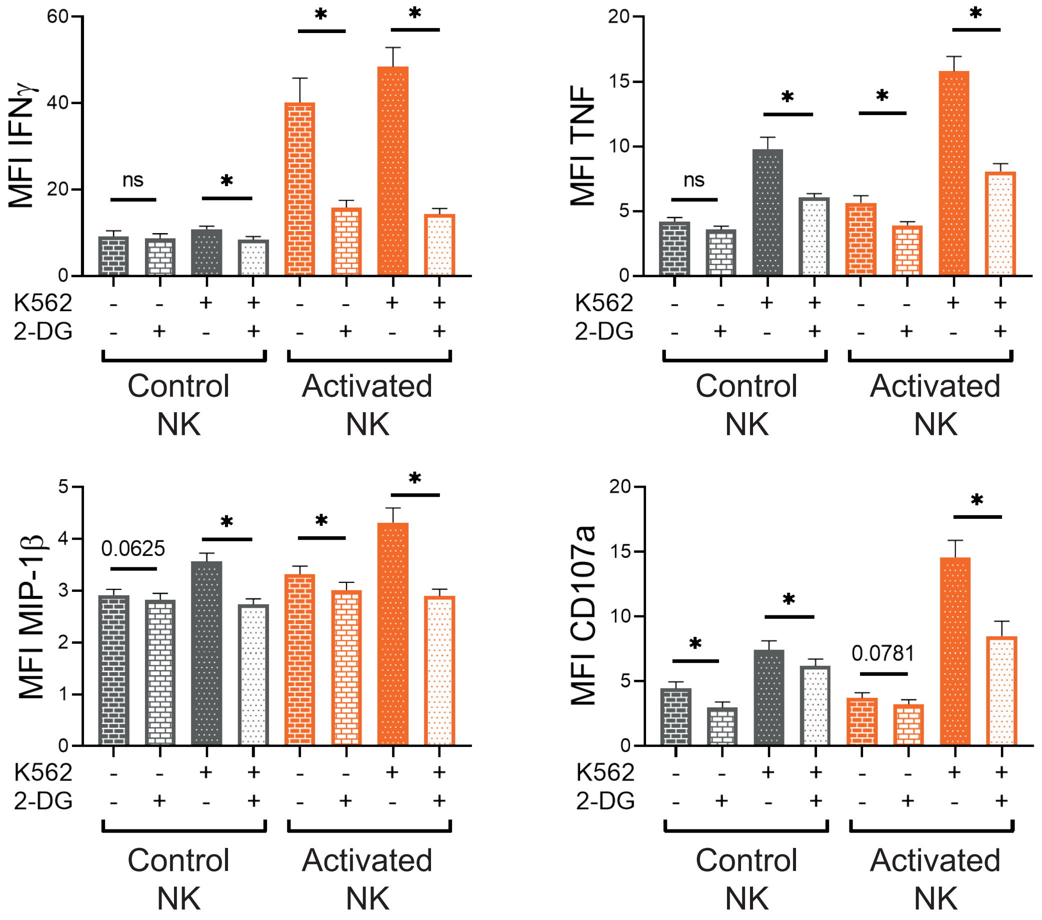

Supplementary Figure 8

Supplementary Figure 8. 2-DG-induced inhibition of cytokine/chemokine production and degranulation of human IL-12/15/18-stimulated NK cells at day 0. Control NK cells and IL-12/15/18-stimulated NK cells (activated NK) were co-cultured with K562 target cells (E:T ratio = 1:1) for 7 hours in the presence and absence of 50 mM 2-DG. Bar graphs showing (A) percentage of positive cells and (B) median fluorescence intensity (MFI) of the cells that are positive for IFNγ, TNF and MIP-1β, or degranulate (CD107a). Means ± SEM are depicted. Statistical analyses were performed using Wilcoxon matched-pairs signed rank test (n = 7). ns = non-significant, *$p<0.05$.

Supplementary Figure 9

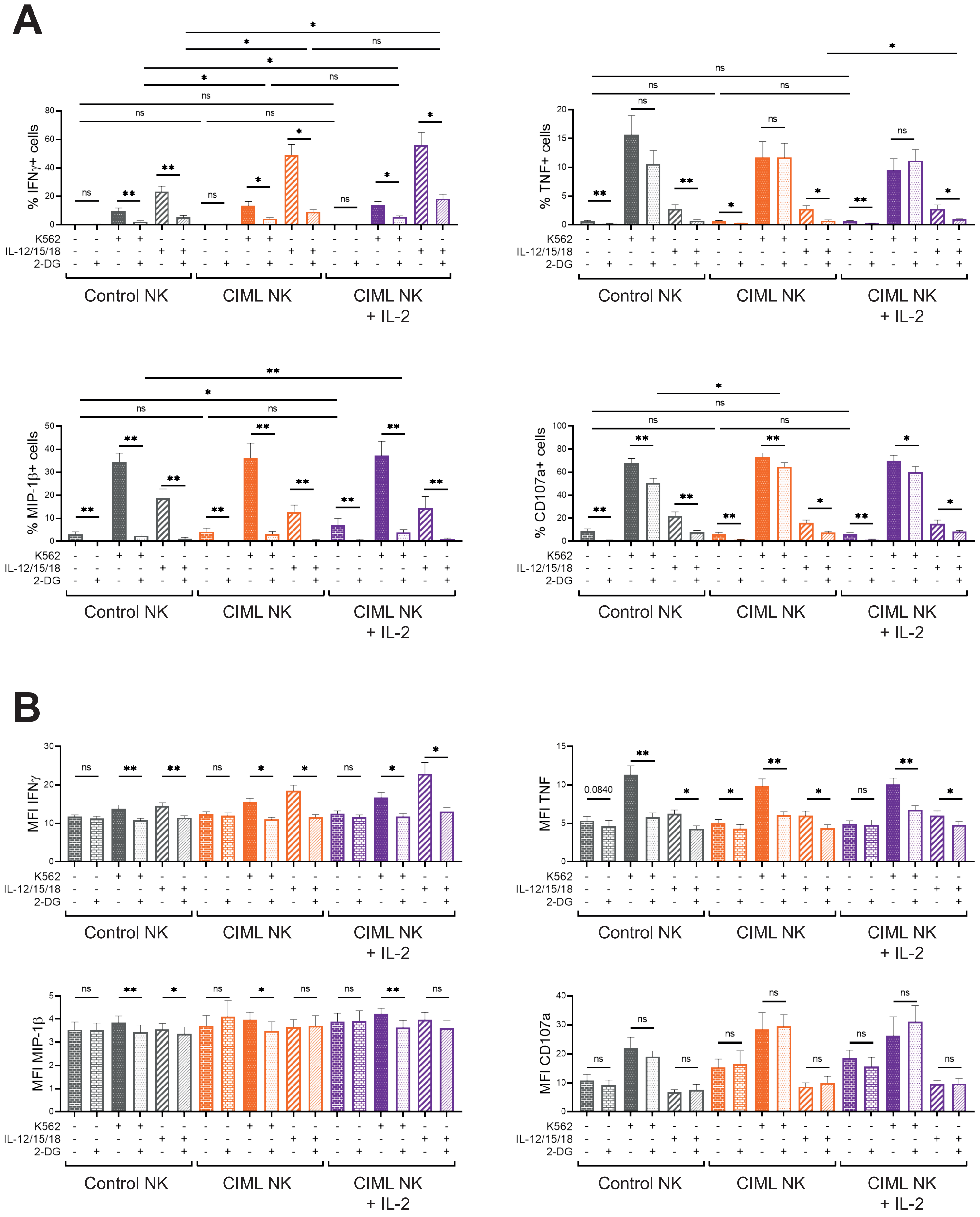

Supplementary Figure 9

Supplementary Figure 9. 2-DG-induced inhibition of cytokine/chemokine production and degranulation of human cytokine-induced memory-like (CIML) NK cells at day 7. Control and activated NK cells cultured for seven days with IL-15 (control NK or CIML NK) or IL-2 (CIML NK+IL-2), were restimulated by co-culturing them with K562 target cells (E:T ratio = 1:1) or restimulated with IL-12, IL-15 and IL-18 (10, 100 and 50 ng/mL, respectively) for 7 hours in the presence and absence of 50 mM 2-DG. Bar graphs showing (A) percentage of positive cells and (B) median fluorescence intensity (MFI) of the cells that are positive for IFNγ, TNF and MIP-1β, or degranulate (CD107a). Means ± SEM are depicted. Statistical analyses were performed using Wilcoxon matched-pairs signed rank test (n = 7-10). ns = non-significant, *$p<0.05$, **$p<0.01$.

Supplementary Figure 10

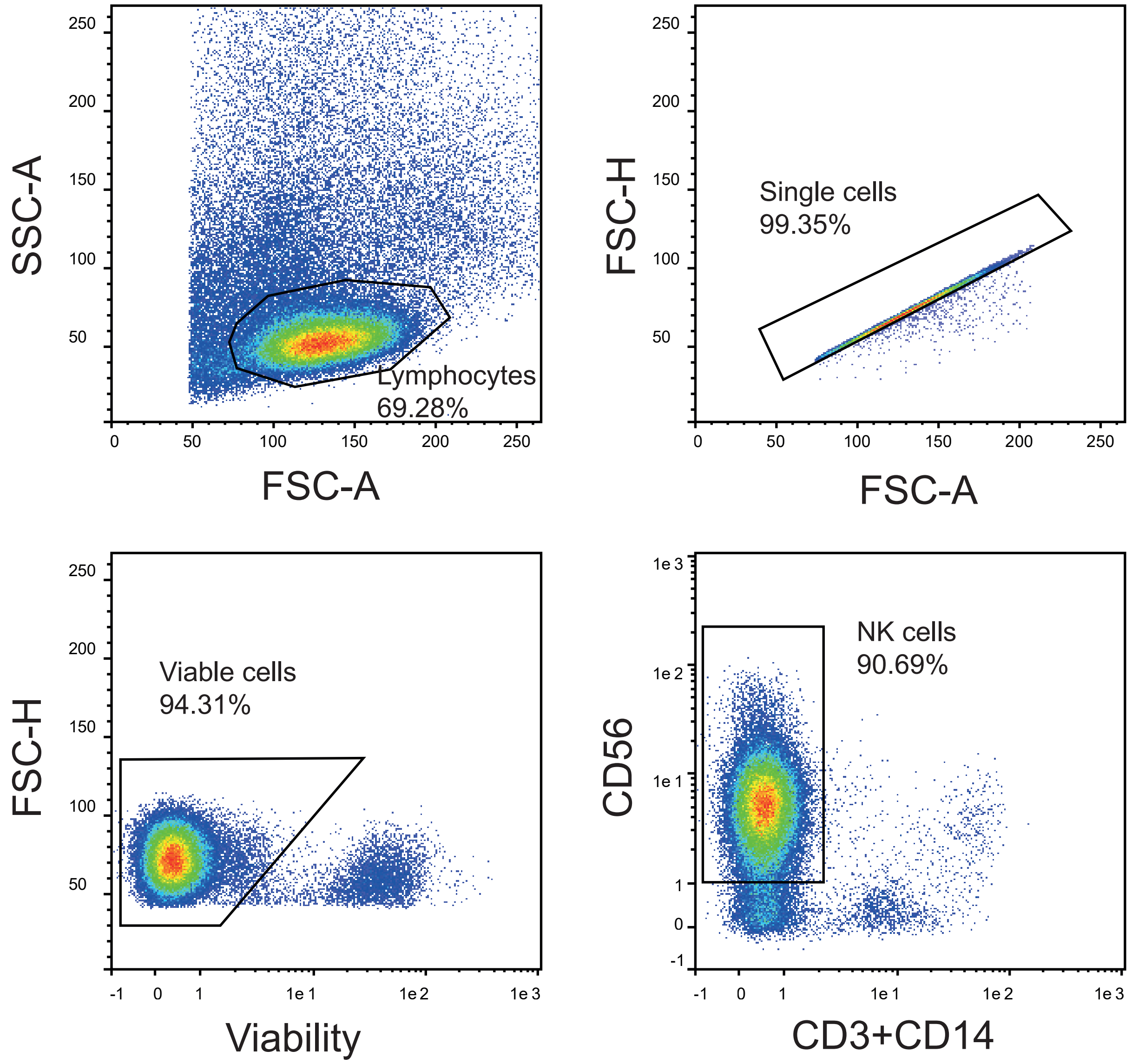

Supplementary Figure 10. Gating strategy of purified NK cells. Lymphocytes were gated attending to forward and side scatter (FSC-A vs. SSC-A) parameters. Then, single cells were identified based on forward scatter (FSC-A vs. FSC-H), and dead cells were excluded with LIVE/DEAD Fixable Near-IR Dead Cell Stain Kit. Finally, NK cells were gated as CD3-CD14- and CD56+.
